# Processing by RNase 1 forms tRNA halves and distinct Y RNA fragments in the extracellular environment

**DOI:** 10.1093/nar/gkaa526

**Published:** 2020-07-01

**Authors:** Gal Nechooshtan, Dinar Yunusov, Kenneth Chang, Thomas R Gingeras

**Affiliations:** Cold Spring Harbor Laboratory, Cold Spring Harbor, NY 11724, USA; Cold Spring Harbor Laboratory, Cold Spring Harbor, NY 11724, USA; Cold Spring Harbor Laboratory, Cold Spring Harbor, NY 11724, USA; Cold Spring Harbor Laboratory, Cold Spring Harbor, NY 11724, USA

## Abstract

Extracellular RNAs participate in intercellular communication, and are being studied as promising minimally invasive diagnostic markers. Several studies in recent years showed that tRNA halves and distinct Y RNA fragments are abundant in the extracellular space, including in biofluids. While their regulatory and diagnostic potential has gained a substantial amount of attention, the biogenesis of these extracellular RNA fragments remains largely unexplored. Here, we demonstrate that these fragments are produced by RNase 1, a highly active secreted nuclease. We use RNA sequencing to investigate the effect of a null mutation of RNase 1 on the levels of tRNA halves and Y RNA fragments in the extracellular environment of cultured human cells. We complement and extend our RNA sequencing results with northern blots, showing that tRNAs and Y RNAs in the non-vesicular extracellular compartment are released from cells as full-length precursors and are subsequently cleaved to distinct fragments. In support of these results, formation of tRNA halves is recapitulated by recombinant human RNase 1 in our *in vitro* assay. These findings assign a novel function for RNase 1, and position it as a strong candidate for generation of tRNA halves and Y RNA fragments in biofluids.

## INTRODUCTION

Cells secrete a variety of RNA molecules to the extracellular environment. These RNAs can be present outside cells in extracellular vesicles (EVs) (1), in lipoprotein particles (2), or as lipid-free particles—either in ribonucleoprotein complexes (3,4) or as free-floating RNAs (5). Interest in the characterization of extracellular RNAs (exRNAs) has risen in recent years, propelled by the discovery that they can be involved in intercellular communication (1,6) and the realization of their diagnostic potential (7). Several studies have cataloged the complement of RNAs present in various extracellular environments, from conditioned tissue culture medium (8–10) to various biofluids such as serum, plasma, urine, saliva and others (11–17). Across those studies, specific fragments that are derived from tRNAs and Y RNAs have consistently been found to be highly abundant.

The most abundant extracellular tRNA fragments bear a striking similarity to tRNA fragments produced intracellularly by angiogenin in response to various stress conditions (18,19). Angiogenin cleaves mature tRNAs at the anticodon loop, producing fragments termed tRNA halves. The sequence of 5′ tRNA halves starts at the 5′ end of the mature tRNA and ends at the anticodon loop. 3′ tRNA halves start at the anticodon loop and end at the 3′ end of the mature tRNA. In stressed cells, intracellular tRNA halves have been shown to repress translation and induce the assembly of stress granules, where stalled translation pre-initiation complexes accumulate (20). They have also been shown to bind cytoplasmic cytochrome *c*, resulting in protection of cells from apoptosis following osmotic stress (21). Notably, intracellular tRNA halves have been implicated in several diseases. They are involved in protection of motor neurons from stress (22), and mutations in angiogenin have been found in several neurodegenerative diseases (23,24). tRNA halves are produced specifically in hormone dependent tumors. They are elevated in estrogen receptor positive breast cancer cell lines and tumor-derived samples, as well as in androgen receptor positive prostate cancer cell lines. Production of these tRNA halves is induced by sex hormones, and leads to enhanced cell proliferation through an as yet uncharacterized mechanism (25). Furthermore, intracellular tRNA half levels are modulated in response to hepatitis virus infection (26) and respiratory syncytial virus (RSV) infection (27). Of note, a tRNA half produced following RSV infection promotes viral replication by posttranscriptional silencing of a host antiviral gene (28).

The source and roles of extracellular tRNA halves are only beginning to unravel. Several studies of mouse sperm have shown that tRNA halves can be delivered from the epididymal epithelium to sperm cells during their maturation in the epididymis. The transfer of tRNA halves and other small RNAs occurs via the extracellular environment, potentially by extracellular vesicles of epididymal source. Following fertilization, these tRNA halves modulate embryonic gene expression in response to paternal diet (29–31). The levels of tRNA halves in biofluids have been shown to respond to various cues, such as ageing and calorie restriction (12) as well as inflammation and viral infection (32). Following these findings, several studies have explored the use of extracellular levels of tRNA halves as biomarkers of prostate (33), breast (14), liver (34) and gastric (35) cancer, as well as clear cell renal cell carcinoma (36,37) and squamous cell carcinoma (38).

Human Y RNAs are a group of 83–113 nucleotide (nt) noncoding RNAs. They were initially identified as components of ribonucleoprotein particles that form autoantigens in systemic lupus erythematosus (39). Intracellular Y RNAs have been implicated in quality control of ribosomal RNA (40) and in DNA replication initiation (41). While intracellular Y RNAs are largely unprocessed in unstimulated cells, they are cleaved following various apoptotic stimuli in a caspase-dependent manner (42). The role of Y RNA fragmentation during apoptosis is still unclear. Y RNAs present in the extracellular environment have been studied in the context of several pathological processes. Y4 RNA in chronic lymphocytic leukemia EVs contributes to formation of a tumor supportive microenvironment by inducing the expression of immunosuppressive PD-L1 and the release of inflammatory cytokines by monocytes. This effect is mediated by endosomal TLR7 signaling (43). Extracellular Y RNA fragments were also shown to modulate the immune response of macrophages to protect cardiomyocytes after ischemia-reperfusion (44). Further evidence for the involvement of extracellular Y RNAs in immunomodulation was provided by Hizir *et al.* (45), who showed that fragments derived from the 5′ and 3′ ends of Y RNAs are upregulated in the serum of atherosclerosis patients. These fragments, complexed with Ro60, induce inflammation and apoptosis in lipid-laden macrophages by activation of the NFkappa-B and caspase 3 pathways. Here too, the effect of Y RNAs is mediated by TLR7 signaling. Changes in serum levels of Y RNA fragments were explored as minimally-invasive diagnostic markers for breast cancer (14) and squamous cell carcinoma (38). Furthermore, Y RNA fragments in serum have been suggested as diagnostic and prognostic markers in coronary artery disease (46).

In spite of their regulatory and diagnostic potential, there is little knowledge about the biogenesis of extracellular tRNA and Y RNA fragments. Here, we show that tRNA halves and distinct Y RNA fragments in the extracellular environment are produced through the action of RNase 1, a broad specificity ribonuclease with proposed role as scavenger of extracellular RNA (47). Our results strongly indicate that tRNAs and Y RNAs are released to the extracellular space as full-length RNAs rather than pre-formed fragments. Following their release, tRNAs and Y RNAs are processed by RNase 1 into specific fragments. Our findings position RNase 1 as a strong candidate for formation of tRNA halves and Y RNA fragments circulating in serum and other biofluids.

## MATERIALS AND METHODS

### Tissue culture

Cells were grown in a humidified incubator at 37°C with 5% CO_2_. Cells were cultured in RPMI 1640 (Thermo Fisher Scientific) supplemented with 10% fetal bovine serum (Thermo Fisher Scientific), penicillin (100 units/ml), streptomycin (100 μg/ml) and glutamine (4 mM) (Thermo Fisher Scientific), or in QBSF-55 serum-free medium (Quality Biological). K562 cell line identity was verified by short tandem repeat profiling, and cells tested negative for the presence of Mycoplasma. Cell viability during culturing and medium conditioning was >95%, as monitored by trypan blue staining.

### Generation of an *RNASE1* null mutant

Stable Cas9 expression was established in the K562 human chronic myelogenous leukemia cell line (ATCC CCL-243) by lentiviral transduction of the pLentiV-Cas9-puro transfer vector. Lentiviral particles were produced by transfection of pLentiV-Cas9-puro (gift from Chris Vakoc Lab, Cold Spring Harbor Laboratory), psPAX2 and pCMV-VSV-G plasmids into the 293FT cell line (Thermo Fisher Scientific). Upon 48 h post-infection (high multiplicity of infection), cells were subjected to puromycin selection to obtain an antibiotic-resistant bulk population.

To generate *RNASE1* mutant cell lines, six sgRNA constructs (Supplementary Table S1) were designed to target regions of the *RNASE1* gene that encode residues important for catalytic activity of the protein. A control cell line carrying wild type *RNASE1* was generated using sgRNA Rosa26_(Mm) (Supplementary Table S1). This sgRNA targets the Rosa26 locus in mice, which is absent from human cells. The sgRNAs were cloned into the lentiviral sgRNA expression vector, LRG2.1 (gift from the Chris Vakoc Lab; available from Addgene). This vector expresses the GFP reporter from the EFS promoter. Each sgRNA construct was infected at single copy (MOI < 0.2) into K562-Cas9 cells and GFP-positive populations were derived by fluorescence-activated cell sorting. Clonal lines were generated by sorting for single GFP-positive cells. The *RNASE1* null clone that was chosen for use in the present work was generated with sgRNA RNASE1_471.58 (Supplementary Table S1). Cells carrying wild type *RNASE1* that were used in this work represent a bulk population of GFP-positive K562-Cas9 cells infected with sgRNA Rosa26_(Mm). All cell lines were characterized by Sanger sequencing, PCR and RT-qPCR (Supplementary Figure S1A, C and D).

### Medium conditioning

For conditioning of RPMI medium, cells were grown in complete RPMI to a density of ∼2.75 × 10^5^ cells/ml. Cultures were spun 10 min at 300 g, the medium was removed, and the cells were gently resuspended in 10 ml of serum-free RPMI. The cell suspension was spun again as above, supernatant was aspirated, and cells were resuspended gently in serum-free RPMI to a density of ∼2.75 × 10^5^ cells/ml. Cells were then returned to the incubator for 24 h, followed by collection of conditioned medium.

Cells grown in QBSF-55 serum-free medium were cultured for at least seven passages before medium conditioning experiments, to ensure full adaptation and normal growth. For medium conditioning in QBSF-55, cells were cultured to a density of ∼2 × 10^5^ cells/ml. Cells were then diluted 15-fold by addition of fresh medium, grown to a density of ∼4 × 10^5^ cells/ml, followed by collection of conditioned medium.

### Isolation of extracellular vesicles and vesicle-depleted medium

Extracellular vesicles were collected essentially as previously described (48). Briefly, conditioned medium was spun twice 10 min at 300 g to remove cells. Then, the supernatant was spun 10 min at 2000 g and 30 min at 10 000 g to remove debris. The supernatant was then spun 70 min at 100 000 g to pellet EVs. The supernatant, containing vesicle-depleted conditioned medium (hereafter referred to as non-EV conditioned medium) was collected. The pellet was resuspended in ice-cold phosphate-buffered saline (PBS; Thermo Fisher Scientific) and spun again 70 min at 100 000 g. All centrifugation steps were carried out at 4°C. Isolated EVs were resuspended in ice-cold PBS and kept overnight at 4°C before RNA extraction.

### RNA preparation

Before RNA isolation, EV suspensions in PBS (250 μl) were treated with a combination of RNase A and RNase T1 before RNA isolation by addition of 10 μl RNase Cocktail (Thermo Fisher Scientific) and incubation at 37°C for 30 min. Non-EV conditioned medium was concentrated at 4°C from 15 ml to 1 ml on 3 kDa cutoff Amicon filters (Millipore-Sigma), as per manufacturer's instructions. Cells for RNA isolation were washed once in 1 ml ice-cold PBS and pelleted by centrifuging 7 min at 300 g at 4°C. Pellets were flash frozen in liquid N_2_ and kept at −80°C until use. RNA was isolated with a mirVana miRNA isolation kit (Thermo Fisher Scientific). The manufacturer's protocol for total RNA isolation was used, with the following exceptions. For EV suspensions and non-EV conditioned medium, RNA isolation was started by addition of two volumes of mirVana Lysis Buffer, and volumes in subsequent steps were scaled up accordingly; organic extraction was spun at 9400 g instead of 10 000 g. RNA intended for sequencing was treated with DNase by using a TURBO DNA-free kit (Thermo Fisher Scientific). Up to 10 μg of RNA was incubated with 2 units of TURBO DNase in 1× DNase buffer (kit-supplied) for 25 min at 37°C. Reactions were inactivated by using the kit-supplied inactivation reagent. RNA was ethanol-precipitated, resuspended in diethyl pyrocarbonate (DEPC)-treated water (Thermo Fisher Scientific) and quantitated using a Qubit fluorometer (Thermo Fisher Scientific). RNA size profiles were analyzed on a Bioanalyzer instrument (Agilent Technologies), using RNA 6000 Pico chips for extracellular RNA samples and RNA 6000 Nano chips for cell RNA samples.

### RNA pretreatments for RNA sequencing

All RNA samples for sequencing were decapped with Cap-Clip acid pyrophosphatase (Cellscript). RNA (up to 1 μg in 21 μl DEPC-treated water) was incubated 2 min at 85°C and transferred to ice for 1 min. 2.5 μl of manufacturer supplied Cap-Clip buffer, 20 units of RNase inhibitor (ANTI-RNase; Ambion) and 2.5 units of Cap-Clip enzyme were added and the reaction (final volume: 25 μl) was incubated for 1 h at 37°C. Following incubation, RNA was phenol-chloroform extracted and ethanol-precipitated. Unless otherwise noted, RNA samples were also treated with T4 polynucleotide kinase (PNK; New England BioLabs). RNA (up to 1 μg in 37.5 μl DEPC-treated water) was combined with 5 μl of manufacturer supplied PNK buffer and 2.5 μl (25 units) PNK. The reaction was incubated 10 min at 37°C, then 5 μl 10 mM ATP was added, followed by 30 min incubation at 37°C. RNA was phenol-chloroform extracted and ethanol-precipitated. Where applicable, the aforementioned PNK treatment was replaced by treatment with the rtStar tRF&tiRNA Pretreatment Kit (Arraystar) as per the manufacturer's protocol. The kit workflow includes 3′ deacylation, followed by end-healing with PNK in the presence of ATP and demethylation by AlkB.

### RNA sequencing

Starting material for RNA sequencing library preparations was 100 ng (before pretreatments as described above). Libraries were prepared using the NEXTFLEX Small RNA-Seq Kit v3 (Bioo Scientific) following the manufacturer's protocol. Reverse transcription reaction bead cleanup was performed in accordance with the manufacturer's alternative protocol without size selection. Libraries were amplified using 16 PCR cycles. Products were size selected on 2% agarose gels to include products larger than the adapter dimer and up to 350 bp (corresponding to RNA inserts up to ∼225 nt). The resulting libraries were sequenced on the Illumina NextSeq 500 platform.

### Analyses of RNAseq data

The primary assembly of the human genome sequence GRCh38.p12 Release 28 was downloaded from GENCODE (https://www.gencodegenes.org/) and used for all analyses in this study. A customized transcript annotation file was constructed as described below. The GENCODE comprehensive gene annotation for the primary assembly of the human genome GRCh38.p12 Release 28 was used as the main source of gene annotations. This annotation does not contain tRNA gene annotations or annotations of *RNY5* and its pseudogenes. Therefore, it was complemented with the annotations of tRNA genes from GtRNAdb (49,50) and with manually prepared annotations of the *RNY5* gene and of its five pseudogenes (*RNY5P1, RNY5P3, RNY5P4, RNY5P5, RNY5P8*). Coordinates of *RNY5* and its pseudogenes were converted from the gene annotation for the previous genome assembly (GRCh37.p13 Release 19) with the LiftOver tool in UCSC Genome Browser (https://genome.ucsc.edu/cgi-bin/hgLiftOver). The resulting customized transcript annotation file is available upon request. Genome indices were generated with STAR v.2.6.1d (51) using our custom transcript annotation file and with *--sjdbOverhang 100* parameter.

Small RNAseq data that were produced in this work were mapped with STAR with the following parameters: *--clip3pAdapterSeq TGGAATTCTC --clip3pAfterAdapterNbases 4 --clip5pNbases 4 --outSAMunmapped Within--outSAMtype BAM SortedByCoordinate --outFilterMultimapNmax 20 --outFilterMismatchNoverLmax 0.05 --outFilterScoreMinOverLread 0 --outFilterMatchNminOverLread 0.85 --outFilterMatchNmin 15 --outMultimapperOrder Random*.

Public small RNAseq data from (52) were used for analysis of tRNA fragments and of tRNA CCA tails in human serum samples. These data were downloaded from SRA accessions SRR7360493, SRR7360492, SRR7360495, SRR7360494, SRR7360487, SRR7360486, SRR7360555, SRR7360554, SRR7360510, SRR7360511, SRR7360508 and SRR7360509. Reads were extracted with fastq-dump v.2.7.0 and mapped with STAR as above, with the following changes to parameters: *--clip3pAdapterSeq TCGTATGCCG --clip3pAfterAdapterNbases 5 --clip5pNbases 0*.

Publicly available long RNAseq data were used to quantify the gene expression levels of catalytically active pancreatic RNases in the K562 cell line. These data were from the ENCODE Project (53) and they were downloaded from SRA accession SRX159822. Reads were extracted with fastq-dump with *--split-3* parameter. Extracted reads were mapped with STAR with the following parameters: *--outSAMtype BAM SortedByCoordinate --outSAMunmapped Within --outFilterType BySJout --outFilterMultimapNmax 20 --alignSJoverhangMin 8 --alignSJDBoverhangMin 1 --outFilterMismatchNmax 999 --outFilterMismatchNoverLmax 0.04 --alignIntronMin 20 --alignIntronMax 1000000 --alignMatesGapMax 1000000 --quantMode TranscriptomeSAM*. Expression levels were calculated with RSEM v.1.2.28 (54) with the following parameters: *--paired-end --forward-prob 0.5 --seed 12345*.

Y RNA and tRNA genes have multiple pseudogenes with highly similar sequences across the genome. In addition, some tRNA genes are also present in multiple identical or highly similar copies. Therefore, our analyses were not limited to uniquely mapping reads, but also included primary read alignments for all multimapping reads. Primary read alignments for unique and multimapping reads were extracted with SAMtools v.0.1.19-44428cd (55). The resulting set of reads was used for all analyses of Y RNAs and tRNAs and of their fragments. Primary alignments were assigned to features in a strand-specific fashion with featureCounts v.1.6.1 (56).

For analysis of Y RNAs, reads that were assigned to Y RNA genes and their corresponding annotated pseudogenes were counted. Primary alignments that were not assigned to any annotated features were further tested for overlap with Y RNA repeats by using BEDTools v.2.17.0 (57). Annotation of Y RNA repeats (annotated as HY1, HY3, HY4 and HY5) was obtained from RepeatMasker (last update date: 2018-08-10). RepeatMasker data were downloaded through the UCSC Table Browser. Reads that mapped to all pseudogenes of a particular Y RNA gene were grouped and counted together. Reads that mapped to all repeats that corresponded to a particular Y RNA gene were also grouped and counted together.

Reads for our analysis of tRNA fragments and of processing of tRNA CCA tails by RNase 1 were selected as outlined below. First, mitochondrially-encoded tRNAs, which differ somewhat in length and structure from their nuclear counterparts (58), were excluded from these analyses. Second, reads were filtered out if they had deletions, insertions or unannotated introns. Third, reads were excluded if they did not have 3′ adapter sequences. Remaining reads were trimmed of adapter sequences before further analysis. Mature human tRNAs are modified to contain a nontemplated CCA tail at their 3′ ends. In the next steps, potential presence of an intact CCA or a processed CC or C tail at the 3′ end was taken into account for the reads whose alignments reached the 3′ end of their corresponding tRNA gene annotations. We refer to those reads as ‘tailed’ hereafter. Adapter-trimmed reads were included into the analysis, if their trimmed length was equal to the sum of alignment matches denoted in the CIGAR string. Tailed reads that contained exactly an intact CCA or a processed CC or C tail sequence at the 3′ end were allowed to be longer than the length of all alignment matches in the CIGAR string by the length of the tail sequence that did not map to the genome. Included reads were further required to not align to pre-tRNA sequences. A read was considered as aligning to pre-tRNA sequence, if its alignment overlapped by at least one nucleotide with either an intron of a tRNA gene or with a sequence outside the annotated mature tRNA. Of note, if a tailed tRNA read mapped to a gene that is followed in the genome by CCA, CC or C, the sequence of the intact CCA or a processed CC or C tail of this tailed read could completely or partially map outside of the corresponding tRNA gene annotation. Such reads were not considered as aligning to pre-tRNA and were included into our analysis.

Reads that qualified for analysis were used for quantification of 5′ and 3′ tRNA halves and for determination of CCA tail status. A read was classified as representing a 5′ tRNA half, if the alignment of such read started no farther than at the fifth nucleotide and ended within nucleotides 29–41 of the corresponding tRNA annotation. A read was considered as representing a 3′ tRNA half, if the alignment started within nucleotides 29–41 and ended within the last five nucleotides of the corresponding tRNA annotation. For analyses presented on Supplementary Figures S5A, F and G, reads that mapped to tRNA genes with identical sequences were grouped together. Detailed data used for producing panels A and F of this figure are presented in Supplementary Table S2.

For analyses of CCA tail status, we counted reads whose alignments reached the annotated end of a tRNA gene and did not have nontemplated nucleotides, or had only C, only CC or the intact CCA sequence following the annotated sequence.

### Northern blots

RNA samples were separated on denaturing 8% polyacrylamide (acrylamide/bis 19:1), 7.8 M urea gels. After electroblotting and UV-crosslinking to nylon membranes (Zeta-probe; Bio-Rad), RNA was detected using end-labeled DNA probes (Supplementary Table S1). Hybridizations were performed in Rapid-hyb buffer (GE Healthcare).

### *In vitro* treatment with recombinant human RNase 1

Vesicle-depleted conditioned medium was pre-incubated for 5 min at 37°C, followed by addition of recombinant human RNase 1 (Bon Opus Biosciences) to the indicated concentration. After 30 min incubation at 37°C, RNA was isolated as described above. RNA was also isolated from vesicle-depleted conditioned medium that was left on ice with no added enzyme. Isolated RNA was analyzed by northern blot.

## RESULTS

### Generation of an *RNASE1* mutant cell line

Pancreatic ribonucleases (RNases) are a family of secreted enzymes. Among human members of this family, RNase 1 has a unique combination of high catalytic activity and robust expression in a wide variety of tissues ((59) and Supplementary Figure S2A). In addition, RNase 1 is present in various biofluids, such as serum, urine, saliva, milk, and seminal plasma (60–62). We reasoned that these traits make it a likely candidate to be the factor responsible for biogenesis of extracellular tRNA halves and Y RNA fragments.

We used CRISPR–Cas9 mutagenesis to target the *RNASE1* gene in the human leukemia cell line K562, which expresses *RNASE1* (Supplementary Figure S2B). We obtained a clone carrying a homozygous 19 bp deletion in the coding sequence of *RNASE1*. This deletion creates a nonsense mutation that is expected to truncate the 128 amino acid wild type mature protein into an N-terminal 45 amino acid polypeptide. The resulting polypeptide would lack two of the three catalytic residues conserved in all catalytically active family members (Supplementary Figure S1A, B).

### The size profile of extracellular RNA changes according to *RNASE1* status

**Figure 1.**
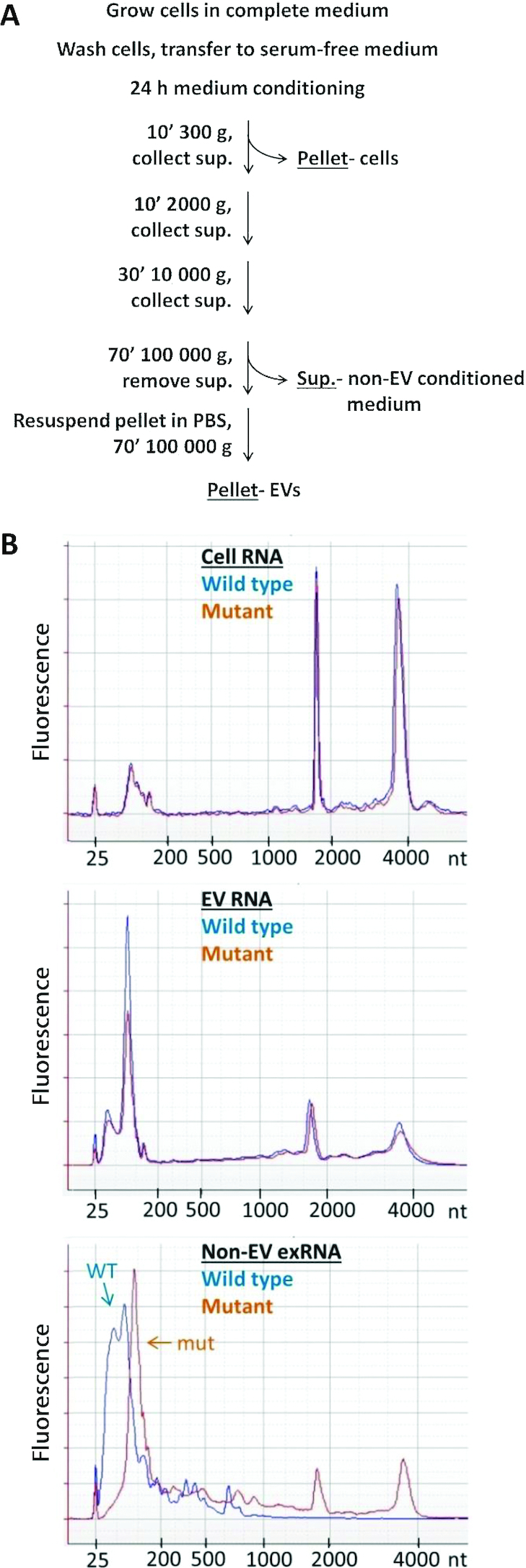
Workflow for collection of samples, and RNA size distribution. (**A**) Procedure for collection of cells, EVs and non-EV conditioned medium. (Sup.) supernatant. (**B**) Representative Bioanalyzer traces of cell RNA, EV RNA and non-EV exRNA from *RNASE1* wild type (WT) and mutant (mut) source cells. The 25 nt peak is the lower size marker.

To assess the gross effect of RNase 1, we compared the size profiles of RNA isolated from *RNASE1* wild type and mutant cells, their extracellular vesicles (EVs) and non-EV conditioned media (Figure 1A). The size profiles of cellular RNA and of EV RNA did not vary substantially between *RNASE1* wild type and mutant source cells. However, the size profile of non-EV extracellular RNA (exRNA) showed a clear shift toward larger sizes in the absence of active RNase 1 (Figure 1B). In addition, RNA yields from non-EV conditioned medium of the mutant were ∼3.6-fold higher (Supplementary Figure S3A), whereas cell RNA yields remained unchanged (Supplementary Figure S3B). These differences suggest that RNase 1 is mainly active in the non-vesicular extracellular environment, in line with RNase 1 being a secreted enzyme that is inhibited inside cells by the endogenous ribonuclease inhibitor (63).

### RNase 1-dependent formation of distinct extracellular tRNA and Y RNA fragments

Based on the differences observed in the size profiles, we decided to sequence non-EV exRNA from wild type and mutant cells. Pancreatic ribonucleases, including RNase 1, leave a 3′ terminal phosphate and a 5′ terminal hydroxyl at the cleavage site (64). These ends are not amenable to ligation by RNA ligases used in commercial RNAseq library preparation kits. In order to achieve an inclusive analysis, input RNA was treated with T4 polynucleotide kinase (PNK) before library construction. Y RNA and tRNA genes both have multiple pseudogenes with highly similar sequences across the genome. In addition, some tRNA genes are also present in multiple identical or highly similar copies. We thus included multimapping reads in the analysis.

**Figure 2.**
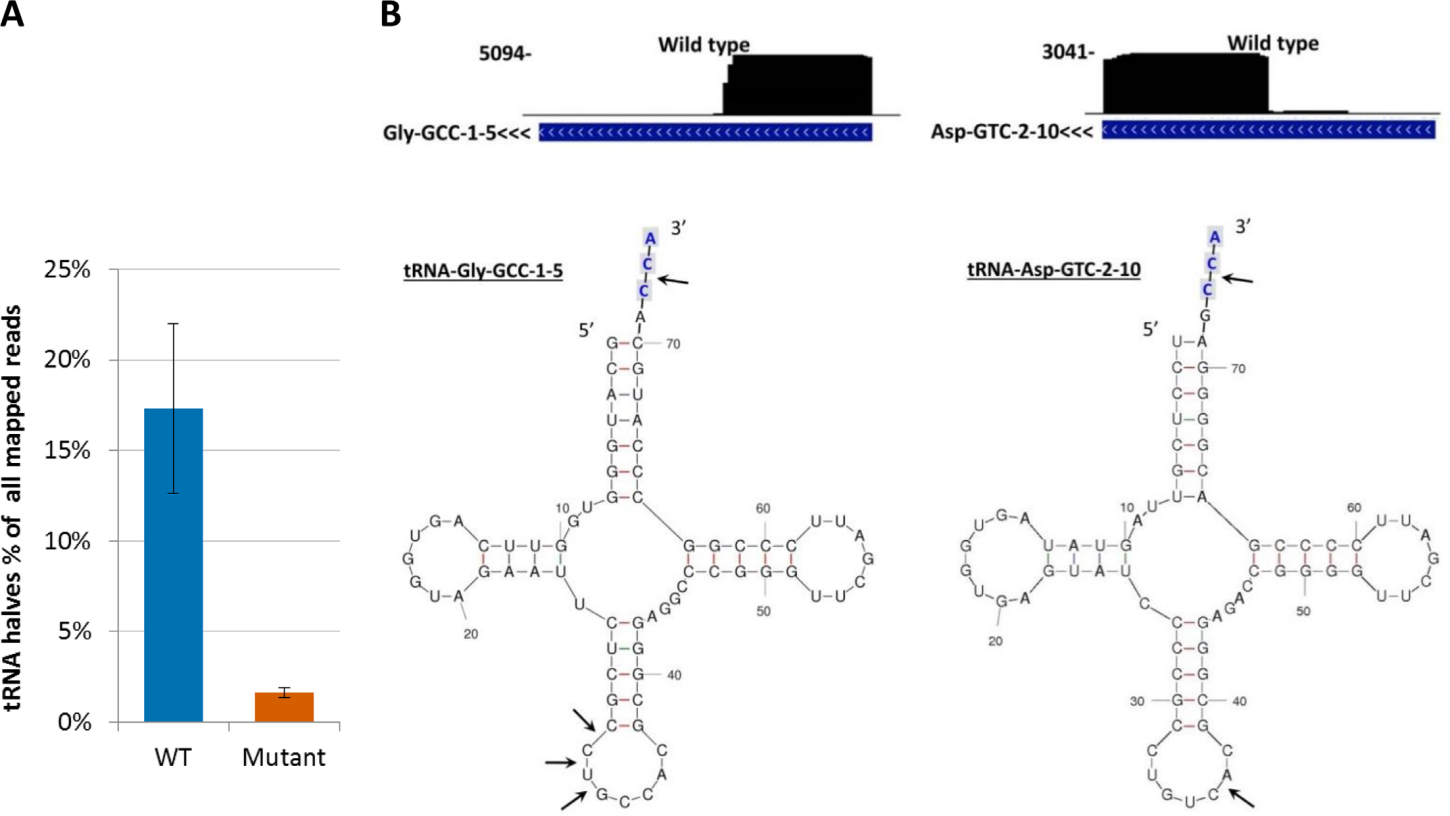
Effect of RNase 1 on formation of tRNA halves. (**A**) Fraction of tRNA halves in RNAseq datasets of non-EV exRNA from *RNASE1* wild type (WT) and mutant cells. Reads considered for tRNA half analysis were required to align to the genome end-to-end after adapter trimming, except that nontemplated additions of C, CC or CCA were allowed after tRNA gene annotation end. See Materials and Methods for definition of halves. Results represent mean ± SD from four independent replicates. (**B**) RNAseq data for representative genes of tRNAs producing the most abundant 5′ halves (tRNA-Gly-GCC) and 3′ halves (tRNA-Asp-GTC). Upper panels: UCSC Genome Browser screenshots depicting RNAseq mapping results of wild type non-EV exRNA. Coverage (vertical axis) is displayed over a tRNA gene annotation track. Arrowheads next to gene names denote direction of transcription. Lower panels: major cleavage sites according to RNAseq data are denoted by arrows on predicted tRNA secondary structures. Predicted structure data are from GtRNAdb (49). Nontemplated CCA tail is shaded.

When we compared non-EV exRNA from *RNASE1* wild type and mutant cells, a clear difference emerged. While reads representing tRNA halves constituted 17.3% of mapped reads in RNA from the wild type, they accounted for only 1.6% of mapped reads in the mutant (Figure 2A). When tRNA genes are visualized on the UCSC Genome Browser, tRNA halves appear to be the main fragments accumulated in non-EV exRNA from the wild type (Figure 2B). The pattern of processing observed here for extracellular tRNAs is analogous to the previously described pattern of processing of tRNAs by angiogenin inside cells (19,25). These results support the hypothesis that RNase 1 is responsible for formation of tRNA halves in the non-EV extracellular compartment.

**Figure 3.**
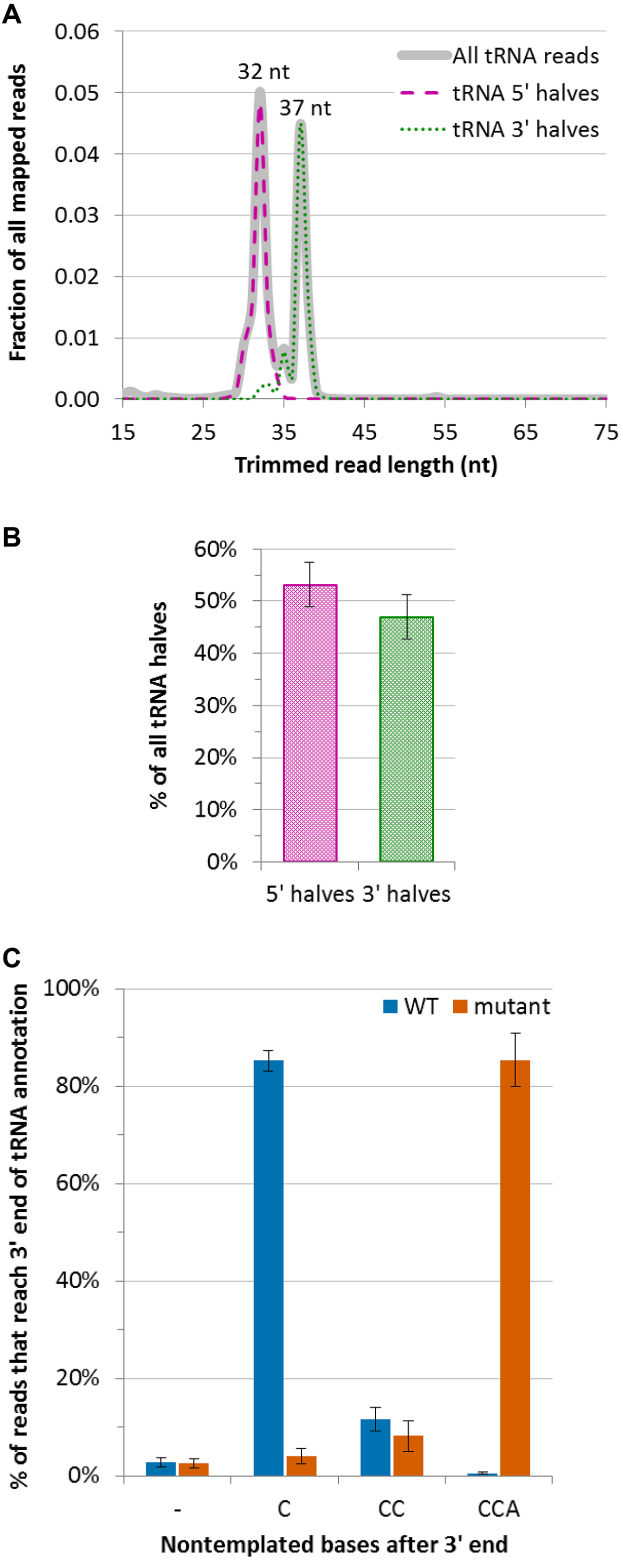
Characterization of 5′ and 3′ tRNA halves and 3′ ends of tRNA in non-EV exRNA. (**A**) tRNA read length distribution in *RNASE1* wild type non-EV exRNA. Length distributions of reads classified as 5′ tRNA halves and reads classified as 3′ tRNA halves are overlaid on the length distribution of all reads that mapped to tRNA. Lengths displayed are after adapter trimming. (**B**) Distribution of reads classified as tRNA halves between 5′ and 3′ tRNA halves in *RNASE1* wild type non-EV exRNA. (**C**) Analysis of CCA tails in reads that reach the 3′ end of tRNA gene annotations in non-EV exRNA. Reads considered for tRNA half and tRNA 3′ end analyses were required to align to the genome end-to-end after adapter trimming, except that nontemplated additions of C, CC or CCA were allowed after tRNA gene annotation end. Results in all panels represent mean from four independent replicates. Error bars in (B) and (C) represent SD.

The size distribution of tRNA fragments in *RNASE1* wild type non-EV exRNA peaked at 32 and 37 nt (Figure 3A), corresponding to 5′ and 3′ halves, respectively. Counts of smaller tRNA-derived fragments (65) were very low, as were counts of reads mapping to mitochondrially-encoded tRNAs (under 0.003% of non-EV exRNA). 5′ halves accounted for 53% of tRNA halves detected, whereas 3′ halves accounted for the remaining 47% (Figure 3B). Interestingly, reads reaching the 3′ end of tRNA in wild type non-EV exRNA generally terminated with a nontemplated C directly after the end of the annotated mature tRNA. Conversely, tRNA reads reaching the 3′ end of tRNAs in *RNASE1* mutant non-EV exRNA mostly ended with an intact nontemplated CCA tail (Figure 3C). This implies that tRNA halves are processed from mature tRNAs with a nontemplated CCA tail, and that this tail is cleaved in an RNase 1-dependent manner. This cleavage is most likely performed by RNase 1, and is consistent with its substrate specificity. Nonetheless, we cannot rule out the possibility that this is done by another nuclease, subsequently to RNase 1-dependent cleavage of the anticodon loop. We also re-analyzed publicly available RNAseq data from human serum samples (52) (Supplementary Figure S4). The results of this analysis show that the pattern of CCA tail cleavage in serum samples is similar to the pattern in our *RNASE1* wild type samples.

The most abundant tRNA halves in our wild type non-EV exRNA were 5′ halves derived from tRNA-Gly-GCC (24.9%) and tRNA-Glu-CTC (17.3%), and 3′ halves derived from tRNA-Asp-GTC (25.8%) (Table 1). 5′ halves of tRNA-Gly-GCC and tRNA-Glu-CTC have been reported to be highly abundant in human plasma, serum, urine and saliva (16), as well as extracellular RNA from glioblastoma primary cells (9) and from mammary epithelial cell lines (8). 3′ halves of tRNA-Asp-GTC have not been reported as abundant in sequencing datasets of exRNA, although both halves of this tRNA are abundant inside breast and prostate cancer cells (25).

**Table 1.** Most abundant tRNA halves in RNAseq of non-EV exRNA from *RNASE1* wild type cells

Fragment	% of all tRNA halves^a^
tRNA-Asp-GTC 3′ half	25.8 ± 2.1
tRNA-Gly-GCC 5′ half	24.9 ± 5.9
tRNA-Glu-CTC 5′ half	17.3 ± 0.7
tRNA-Glu-TTC 3′ half	7.3 ± 1.6
tRNA-Glu-CTC 3′ half	6.7 ± 2.5
tRNA-Val-TAC 3′ half	5.5 ± 1
tRNA-Lys-CTT 5′ half	2.8 ± 1.1
tRNA-Val-CAC 5′ half	2.4 ± 0.5
tRNA-Gly-CCC 5′ half	1.6 ± 0.4
tRNA-Val-TAC 5′ half	1.5 ± 0.3

^a^Mean ± SD of four independent replicates.

**Figure 4.**
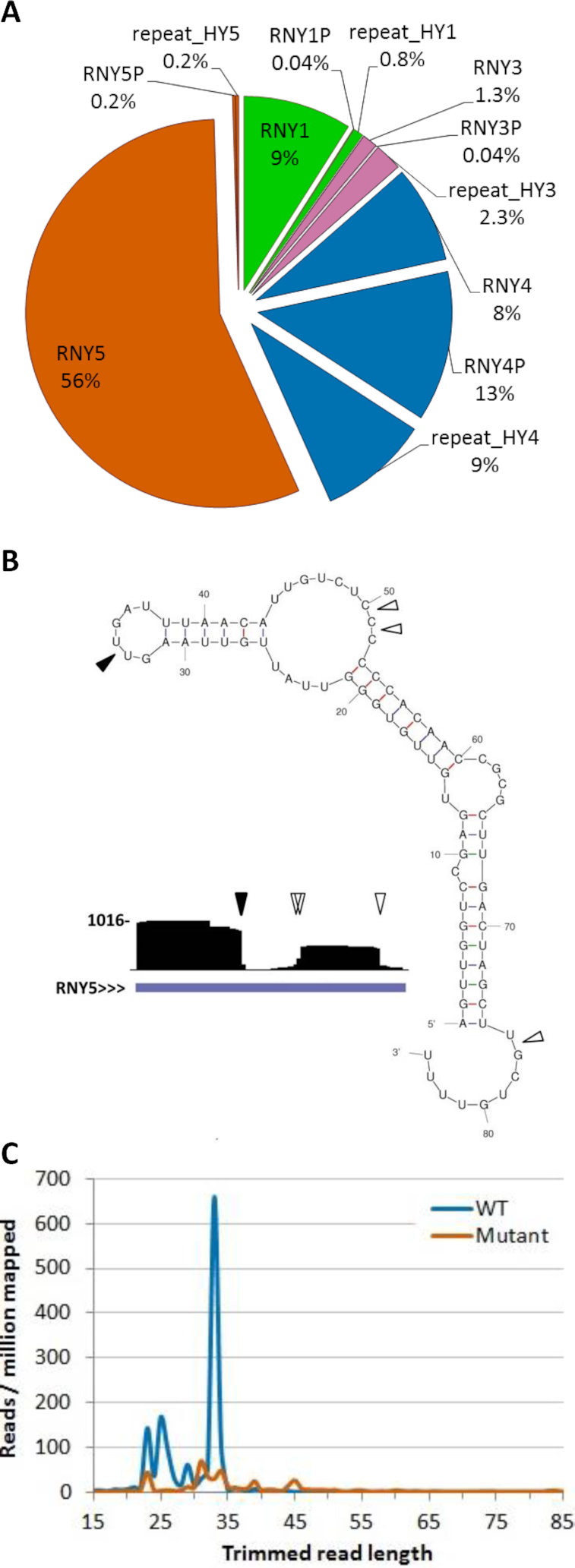
RNAseq analysis of RNY5 in non-EV exRNA. (**A**) Distribution of reads mapping to Y RNAs between Y RNA genes, their pseudogenes and Y RNA - associated repeats in *RNASE1* wild type non-EV exRNA. Reads mapping to all pseudogenes of a particular Y RNA gene were aggregated. Reads mapping to all repeats associated with a particular Y RNA gene were also aggregated. Locations of Y RNA - associated repeats were retrieved from RepeatMasker. (**B**) UCSC Genome Browser screenshot of reads mapping to *RNY5* in *RNASE1* wild type non-EV exRNA, with cleavage points depicted on RNY5 structure. Coverage (vertical axis) is displayed over a RefSeq gene annotation track. Full arrowhead indicates cleavage point of 5′ fragment. Open arrowheads indicate cleavage points of 3′ fragment. Structure is based on (69). (**C**) Length distribution of adapter-trimmed reads that map to *RNY5* in *RNASE1* wild type and mutant non-EV exRNA. Note that full-length RNY5 is poorly captured by small RNA sequencing, probably due to stable secondary structure (see text). Results in (A) and (C) represent the mean of four independent replicates.

Among reads mapping to Y RNA genes, RNY5 reads represented 56% (Figure 4A). In wild type non-EV exRNA, we observed a prominent 33 nt fragment derived from the 5′ side of the RNY5 primary transcript, as well as a shorter 3′ fragment. These fragments were nearly absent in datasets from the mutant, where RNY5 was represented by fewer, less distinct fragments (Figure 4B, C). These results indicate that in addition to its role in formation of extracellular tRNA halves, RNase 1 is also involved in formation of distinct extracellular Y RNA fragments.

### Validation of RNAseq results by northern blots

Small RNA sequencing suffers from several shortcomings. First, the reverse transcriptase enzyme used for synthesizing cDNA from adapter-ligated RNA has low processivity and stops synthesis upon reaching stable structures in the RNA (66). Additionally, the advance of reverse transcriptase along the template is hindered by various base modifications (67). Since tRNAs are characterized by both stable secondary structure and abundant base modifications (68), full-length mature tRNAs are inefficiently cloned to RNAseq libraries. Conversely, tRNA halves have less secondary structure and a lower number of modifications per molecule. Hence, they can be more efficiently cloned to RNAseq libraries. Furthermore, mature tRNAs may be aminoacylated on their 3′ end, which would preclude their cloning to an RNAseq library.

**Figure 5.**
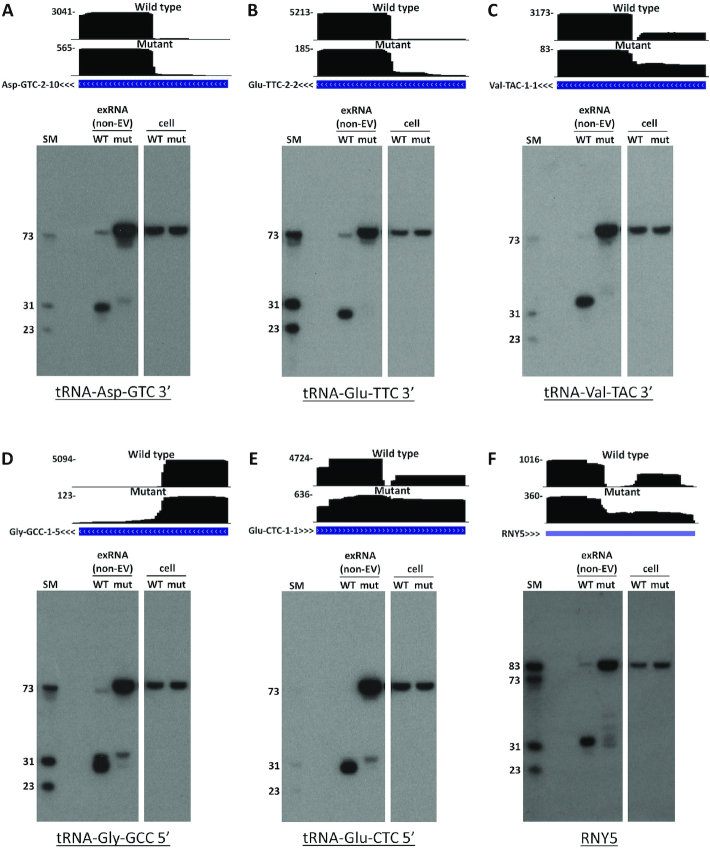
Northern blots of tRNAs and RNY5 from wild type (WT) and *RNASE1* mutant (mut) non-EV exRNA and cell RNA. Probe targets are indicated under each blot. exRNA lanes were loaded with RNA from 2.5 ml of EV-depleted conditioned medium (3.5 ml for RNY5). Cell RNA lanes were loaded with 500 ng total RNA from cells that were used for conditioning of serum-free RPMI (700 ng for RNY5). UCSC Genome Browser screenshots for representative tRNA genes (**A–E**) or for RNY5 (**F**) are presented above each blot. Coverage (vertical axis) is displayed over a tRNA (panels A-E) or RefSeq (panel F) gene annotation track. Arrowheads next to gene name denote transcription direction. (SM) end-labeled RNA size marker. Note that sizing is approximate due to incomplete denaturation of stable secondary structures. This is particularly pronounced for 3′ tRNA halves. Full-length mature tRNAs analyzed here are 74–76 nt long (including the CCA tail). Full-length RNY5 is 84 nt long. Note that most full-length tRNAs and Y RNAs are inefficiently cloned into sequencing libraries (see text).

Northern blots are not subject to the above biases. Therefore, we proceeded to validate and expand the results obtained with RNAseq using this approach. Northern analysis of several genes (Figure 5A–E) showed that tRNAs in cell RNA were full-length regardless of *RNASE1* status. Importantly, non-EV exRNA from the *RNASE1* mutant contained almost exclusively the full-length tRNA, with a small amount of fragment present for some genes. Conversely, non-EV exRNA from wild type contained almost exclusively fragments corresponding in size to tRNA halves. These results show that RNase 1 forms extracellular tRNA halves, corroborating our RNAseq findings. Furthermore, the results suggest that extracellular tRNA halves are made in the extracellular environment from full-length mature tRNAs. Notably, the small amount of full-length tRNA present in wild type non-EV exRNA was slightly shorter than in the mutant and in cells. This is likely due to cleavage within the CCA tail, as observed in RNAseq results.

Our RNAseq data suggest that most tRNAs are present in non-EV exRNA as either mostly 5′ or mostly 3′ halves (Supplementary Figure S5A). Imbalanced 5′/3′ representation has been observed in previous exRNA sequencing studies (8,12,17). We used northern analysis in order to understand whether such imbalance is a result of incomplete representation in sequencing libraries or a genuine biological phenomenon. We chose to analyze both 5′ and 3′ halves of tRNA-Gly-GCC, which showed only 5′ halves on RNAseq and of tRNA-Asp-GTC, which showed only 3′ fragments on RNAseq (Supplementary Figure S5B). For both genes, we found that substantial signal existed for both 5′ and 3′ halves. This means that at least for some genes, both halves remain intact in non-EV exRNA even if this is not evident from RNA sequencing.

Similarly to tRNAs, Y RNAs form stable secondary structures (69) that can impede their cloning to RNAseq libraries. Indeed, our RNAseq results of mutant non-EV exRNA showed a decrease in the number of reads that correspond to fragments of RNY5, which is the most abundantly represented Y RNA in our RNAseq datasets. This decrease was not accompanied by an increase in the number of full-length RNY5 reads (Figure 4C). Therefore, we also performed a northern analysis for RNY5 (Figure 5F). This analysis showed that RNY5 was full-length in cells and mostly full-length in mutant non-EV exRNA. Conversely, in wild type non-EV exRNA, full-length RNY5 signal was very low, whereas a 5′ fragment was abundant. The size of this fragment agreed with the 33 nt fragment size observed in RNAseq. These findings support the notion that Y RNA fragments are produced by RNase 1 from full-length transcripts in the extracellular environment.

### **tRNA modifications contribute to imbalanced 5**′**/3**′ **half representation in RNAseq data**

tRNAs are highly modified (68) and may be aminoacylated. Since these factors can be expected to impede cloning to RNAseq libraries, we hypothesized that they may be an underlying reason for imbalanced 5′/3′ half representation of individual tRNAs. To address this question, we repeated our RNA sequencing with added steps for RNA deacylation followed by base demethylation using AlkB (70). Analysis of these datasets revealed a significant increase in tRNA half detection (Supplementary Figure S5C). When we analyzed the overall balance between 5′ and 3′ tRNA halves, we did not detect significant differences (Supplementary Figure S5D). This suggests that 5′ and 3′ tRNA halves are affected by the additional treatments to a similar extent. If aminoacylation were a substantial obstacle to cloning of 3′ tRNA halves, an increase in the detection of these fragments, specifically, would be expected. Since this was not observed, it appears that deacylation does not contribute appreciably to the representation of tRNA halves in exRNA. We then analyzed individual tRNA genes, and found that for some of them, the 5′/3′ imbalance was reduced (Supplementary Figure S5E). Globally, the percentage of individual tRNAs that have less than 10:1 imbalance between their two halves increased from 18.4% to 40.6% (Supplementary Figure S5F, G). This indicates that RNA demethylation can decrease the 5′/3′ imbalance of individual tRNAs in RNAseq libraries by facilitating broader detection of tRNA halves. Taken together, our northern analysis and our results from sequencing after RNA demethylation suggest that both parts of tRNA molecules cleaved by RNase 1 can be preserved in the extracellular environment.

### Recombinant RNase 1 recapitulates tRNA half formation in conditioned medium

**Figure 6.**
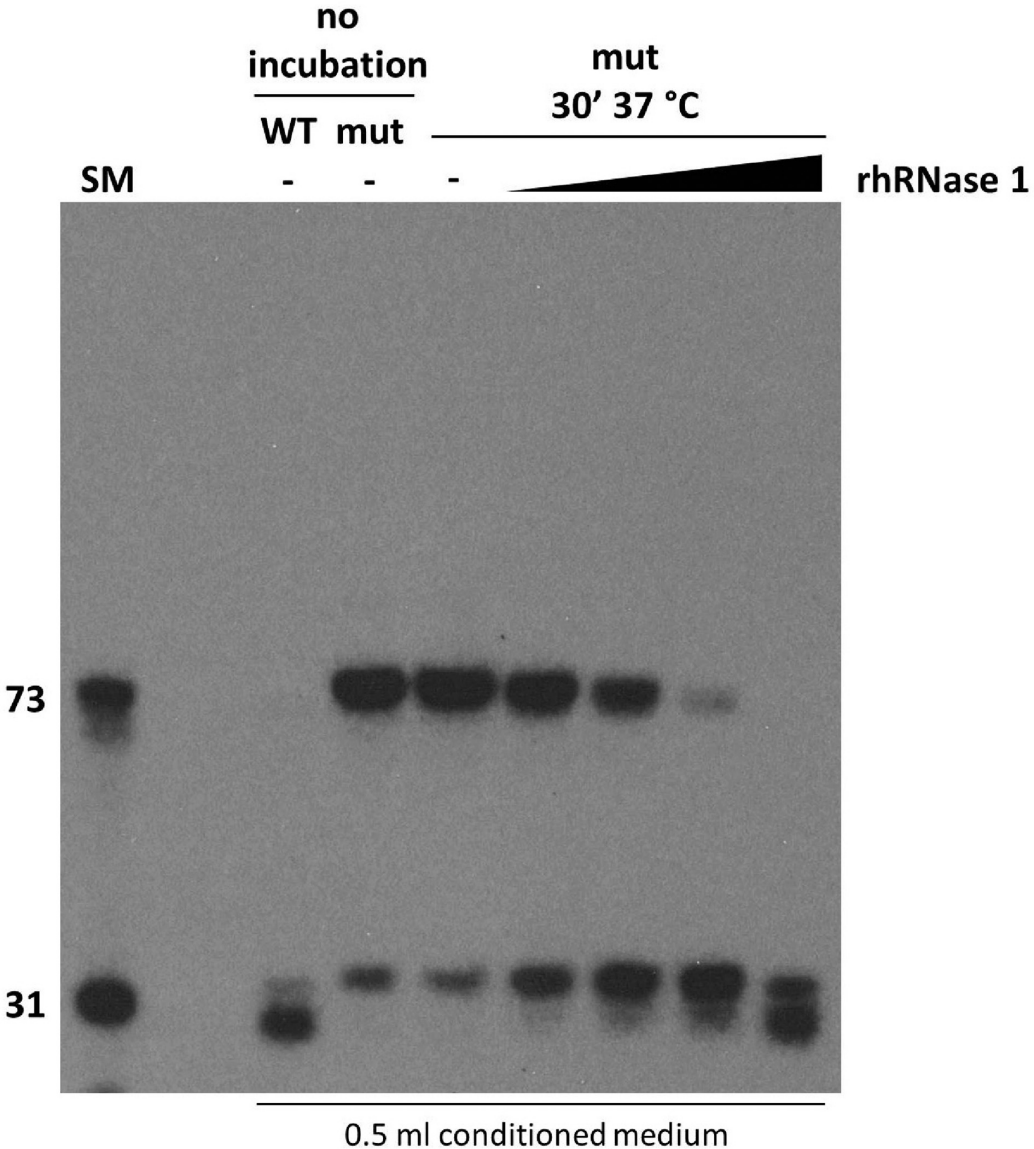
Recombinant human RNase 1 recapitulates formation of tRNA halves *in vitro*. Where indicated, EV-depleted conditioned medium from *RNASE1* wild type (WT) or mutant (mut) cells was incubated at 37°C with recombinant human RNase 1 (rhRNase 1). Recombinant enzyme was added to a final concentration of 0.5, 1.5, 5 or 15 ng/ml. RNA was isolated and a northern blot was performed with a probe against the 5′ half of tRNA-Gly-GCC. (SM) end labeled RNA size marker.

In order to further verify that RNase 1 is capable of producing tRNA halves outside cells, we added recombinant human RNase 1 to vesicle-depleted conditioned medium from *RNASE1* mutant cells. We then isolated RNA from the medium and performed a northern blot using a probe for the 5′ half of tRNA-Gly-GCC. As shown in Figure 6, untreated medium contains mainly full-length tRNA and only a small amount of fragments. Addition of recombinant human RNase 1 caused a dose-dependent decrease in full-length tRNA and concomitant formation of fragments corresponding to tRNA halves. Furthermore, remaining full-length tRNA becomes slightly shorter in the presence of RNase 1. This is similar to northern results of non-EV exRNA from *RNASE1* wild type cells (Figure 5A-D), and likely reflects cleavage in the nontemplated CCA tail. These results further demonstrate that RNase 1 is capable of forming tRNA halves.

### Presence of extracellular tRNA halves is independent of stress-induced intracellular tRNA cleavage

In this study, medium conditioning was performed in serum-free RPMI medium in order to avoid the abundant RNases and bovine RNA present in fetal bovine serum. However, a previous study reported that various stress conditions, including serum deprivation, can lead to angiogenin-mediated formation of intracellular tRNA halves (19). If released, these fragments could serve as a source of extracellular tRNA halves. To address this concern, we repeated our RNAseq study with QBSF-55, a serum-free medium capable of supporting long-term K562 growth. As shown in Supplementary Figure S6A, the results from QBSF-55 conditioned medium were similar to those with serum-free RPMI (compare to Figure 2A). High levels of tRNA halves were present in non-EV exRNA from wild type cells, whereas they were present in low levels in exRNA from *RNASE1* mutant cells. Furthermore, tRNA halves were undetectable in northern analysis of cell RNA isolated from cells after 24 h in serum-free RPMI (Figure 5A-E and Supplementary Figure S6B). Taken together, these results make it unlikely that tRNA halves observed in non-EV exRNA are produced inside cells due to stress in our experimental setup.

## DISCUSSION

Studies of RNA in the extracellular environment have exposed an unexpected abundance of small noncoding RNAs in this compartment. While the function of extracellular small RNAs gets a considerable amount of study, the mechanisms that bring about their abundant presence remain largely unexplored. In this work, we aimed to characterize the biogenesis of two highly abundant groups of noncoding RNAs in the extracellular space- namely, tRNA halves and Y RNA fragments. By targeting the *RNASE1* gene, which gives rise to the highly active and widely present enzyme RNase 1, we showed evidence that tRNAs and Y RNAs are released by cells as full-length precursors. These precursors are then cleaved in the extracellular environment by RNase 1 to yield tRNA halves and distinct Y RNA fragments.

Since angiogenin is known to produce intracellular tRNA halves under stress (18,19), it has been suggested that it may also be responsible for formation of tRNA halves in the extracellular space. Our findings suggest that RNase 1, a member of the same family of enzymes, operates in the extracellular space in an analogous manner to angiogenin inside cells, by cleaving tRNAs in the anticodon loop. Furthermore, our data show that tRNAs in non-EV exRNA from *RNASE1* wild type cells are cleaved at their CCA tail, likely by RNase 1. Cleavage at the CCA tail has also been shown for angiogenin (71). Notably, RNase 1-dependent cleavage of the CCA tail occurs between the two cytidine residues of the CCA tail, whereas angiogenin cleaves between the second cytidine and the adenosine, probably due to the substrate preference of this enzyme (71). Several recent studies claim that tRNA halves are produced in cells even in the absence of angiogenin (72,73), or that cleavage of tRNAs at both anticodon loop and CCA tail is performed in the absence of angiogenin (74). Further work is needed to establish whether RNase 1 is involved in formation of intracellular tRNA halves in response to stress.

While processing of tRNAs by RNase 1 gives rise to extracellular fragments that are similar to those produced inside cells, an important difference can be noted. The presence of tRNA fragments outside cells is independent of intracellular stress-induced tRNA cleavage. This is supported by several lines of evidence. First, our northern analysis of total RNA from cells incubated in serum-free RPMI for the duration of medium conditioning shows no evidence of stress-induced tRNA cleavage (Figure 5A–E and Supplementary Figure S6B). Second, tRNA halves are abundant in non-EV exRNA from cells grown in the serum-free medium QBSF-55, which supports long-term growth of K562 cells (Supplementary Figure S6A). In line with this, a previous study, using defined media in a different experimental setup (8) showed that the presence of tRNA halves derived from tRNA-Gly-GCC and tRNA-Glu-CTC in EV-depleted conditioned medium is not a result of stress from serum deprivation. Finally, tRNA halves are abundant in biofluids from healthy individuals, further supporting the notion that extracellular tRNA halves can be produced in the absence of cell stress.

Various studies of extracellular RNA indicate that the vast majority of tRNA halves detected are 5′ halves (8,12,15,17). In our datasets, however, 3′ halves are well represented. 47% of all tRNA halves detected are 3′ halves (Figure 3B), and among the 10 most abundant tRNA halves, four are 3′ halves (Table 1). While this difference from previous studies could be a specific attribute of K562 cells, we suggest a different explanation. Since RNase 1 leaves a 3′ phosphate (usually a 2′-3′ cyclic phosphate) and a 5′ hydroxyl at the cleavage site, the resulting fragments are not amenable to adapter ligation by RNA ligases used in library preparation protocols. Considering this characteristic of RNase 1, we decided to treat our RNA samples with PNK before library preparation. However, most previous RNAseq studies describing tRNA halves do not report using this step. To assess the importance of PNK treatment, we also prepared RNAseq libraries omitting PNK treatment. These libraries display a sharp decrease in representation of 3′ tRNA halves compared to libraries from PNK treated RNA (Supplementary Figure S7A). We also re-analyzed publicly available human serum RNA sequencing datasets prepared with and without PNK treatment (52). This analysis showed a rise in 3′ tRNA half representation after PNK treatment (Supplementary Figure S7B). It is unclear why 3′ fragments are more affected by this problem. It is possible that a certain phosphatase activity removes 3′ phosphates from cleaved RNAs whereas the complementary 5′ end-healing activity is not available to the same extent. Additionally, as our data show, most 3′ halves are the result of two cleavages by RNase 1 (at the anticodon loop and at the 3′ CCA tail). Conversely, 5′ fragments are the product of a single cleavage. Thus, 5′ halves require end healing only on their 3′ end, whereas 3′ halves require end healing on both 5′ and 3′ ends, which may be less efficient.

Although the overall representation of 5′ and 3′ tRNA halves in our PNK-treated non-EV exRNA datasets is similar (Figure 3B), the majority of individual tRNAs appear as either mostly 5′ or mostly 3′ halves (Supplementary Figure S5A). However, our northern analysis showed that at least for two abundant tRNAs, both 5′ and 3′ halves are present in exRNA despite sharply imbalanced representation on RNAseq (Supplementary Figure S5B). A recent study (31) also reported northern detection of tRNA fragments that were not detected by RNAseq. We hypothesized that at least two factors could contribute to 5′/3′ biased tRNA half representation. First, tRNAs are heavily modified (68), and some of these modifications hinder reverse transcription (67). Since cloning of RNAs to small RNA sequencing libraries requires end-to-end reverse transcription, even the presence of one such modification per tRNA half could interfere with cloning. Second, tRNAs may have an amino acid covalently attached to their 3′ end, which would block adapter ligation. We attempted to minimize these sources of bias by adding steps to demethylate and deacylate the RNA before library construction. Indeed, our analysis showed that the 5′/3′ imbalance of individual tRNAs is reduced by these steps (Supplementary Figure S5G and compare Supplementary Figures S5A and S5F), probably mainly by demethylation. Our northern results, combined with results of demethylated RNA sequencing, indicate that both tRNA halves made by RNase 1 may be preserved in the extracellular RNA of K562 cells. Careful assessment is needed in order to establish whether this may apply to other environments rich in RNase 1, such as biofluids. Furthermore, our findings do not exclude the possibility that for some tRNAs, one of the halves is bound in a complex that protects it from degradation whereas the other half is efficiently degraded.

Our results also show that some tRNAs are not affected by demethylation and deacylation, and their representation on RNAseq remains imbalanced (Supplementary Figure S5E). In that regard, it should be noted that dozens of different types of RNA modifications are known (75), and only some of them are methylations. Furthermore, AlkB, used in this work to demethylate RNA, only removes several types of base methylations (70). Thus, it is very likely that many modifications that hinder reverse transcription remain after treatment with AlkB. Nevertheless, our findings clearly show that standard sequencing methods employed to detect tRNA fragments leave many tRNA halves undetected. A combination of 3′ deacylation, modification removal, end healing, and specialized library preparation procedures (76) is anticipated to substantially improve detection of tRNA halves. These improvements may also have implications for biomarker discovery.

A small subset of genes dominates the category of tRNA halves in our RNAseq datasets. 5′ halves of tRNA-Gly-GCC and tRNA-Glu-CTC, and 3′ halves of tRNA-Asp-GTC, are extremely abundant, together accounting for 68% of all tRNA halves detected (Table 1). The high abundance of specific tRNA halves in the extracellular environment may be dictated by several factors. First, some tRNAs can be more highly expressed than others. Second, some tRNAs may be exported more efficiently. Finally, some tRNA halves may be more stable than others, either due to binding by a protein factor, or due to intrinsic properties of the RNA molecule. Earlier reports have highlighted 5′ halves of tRNA-Gly-GCC and tRNA-Glu-CTC as highly abundant (8,9,16). A recent report (5) showed that these tRNA halves can form homo- and hetero-dimers, and that such dimerization contributes to their stability. Unlike tRNA-Gly-GCC and tRNA-Glu-CTC, tRNA-Asp-GTC has not been reported to give rise to high-abundance tRNA halves. However, the expression of specific tRNA genes can vary substantially between different cell types (77). Furthermore, certain cancer cells were shown to upregulate specific tRNAs (78). Thus, it is possible that K562 cells overexpress tRNA-Asp-GTC. Such overexpression may explain the abundance of fragments derived from tRNA-Asp-GTC in our datasets. Technical considerations may also underlie the abundant representation of these fragments. Our library preparation procedure used a kit that employs four randomized nucleotides at the ends of RNA adapters. This is meant to reduce biases typical to ligation-based library preparation procedures, and thus may enhance detection of fragments that are poorly cloned when other kits are used.

tRNAs are known to give rise to various fragments different from halves. These fragments can be produced as a result of cleavage at sites other than the anticodon loop, by nucleases other than angiogenin (79). As evident from the size distribution of reads mapping to tRNAs, the vast majority of these reads in our samples have lengths typical to tRNA halves. Other sizes do not appear to be present in appreciable amounts (Figure 3A). It is possible that the nucleases that produce tRNA fragments other than halves are inactive under the conditions we tested. Alternatively, these nucleases may be limited to intracellular activity and the resulting fragments may remain inside cells. Given the wide substrate specificity of RNase 1, this enzyme could be expected to give rise to a wider array of products than observed for tRNAs and Y RNAs. It appears likely that bound proteins, base modifications and/or RNA structure limit the spectrum of fragments that can be produced from these RNAs.

In spite of our findings showing that RNase 1 is responsible for formation of tRNA halves and Y RNA fragments, some of them are still observed in non-EV exRNA from the mutant (Figure 5). These fragments could have several sources. First, all members of the pancreatic RNase family possess a signal peptide, and could thus be present and active in conditioned medium, albeit to a lesser extent than RNase 1. RNases from other families could also be present and produce some fragments. Second, K562 cells in our study were grown in complete medium containing bovine serum RNases. Although cells were carefully washed before medium conditioning, some bovine RNase could remain in the medium. Lastly, fresh RPMI medium, as well as QBSF-55 medium may contain contaminating RNase activity. Notably, fragments in exRNA from the mutant tend to be larger than those present in exRNA from wild type cells. It is possible that bases at the middle of the anticodon loop are the most susceptible to attack, and any pancreatic RNase activity present preferentially cleaves them before proceeding to cleave further towards the stem. This phenomenon is also evident in our *in vitro* processing assay (Figure 6), where products of cleavage by lower concentrations of RNase 1 run larger than those produced in the presence of higher concentrations of enzyme. The difference in size between 3′ tRNA halves in wild type *vs*. mutant non-EV exRNA may also be explained by differences in the level of cleavage in the CCA tail.

Our *in vitro* processing assay shows that recombinant RNase 1 is able to recapitulate formation of tRNA halves in cleared conditioned medium from the mutant (Figure 6). The concentration of RNase 1 that gives the most similar processing pattern to that found in *RNASE1* wild type non-EV exRNA is 15 ng/ml. Average serum concentrations of RNase 1 in healthy individuals have been reported to be 189 ng/ml (80) and 400 ng/ml (81). While these concentrations are substantially higher, serum may differ from cleared conditioned medium in ionic strength, pH, protein factors present and redox potential. These factors may affect processing efficiency. More importantly, blood is a dynamic system in which there is constant addition of new substrate, whereas our *in vitro* processing system is static in that regard, since it does not contain cells.

While the catalytic mechanism of RNase 1 has been studied in detail, the role of this enzyme remains less well defined. Several studies have suggested RNase 1 as a scavenger of extracellular RNA, involved in immunomodulation and modulation of coagulation (82–84). The results of our study suggest a novel role for RNase 1 as a factor involved in production of abundant extracellular tRNA halves and distinct Y RNA fragments. Clearly, biofluids are a much more complex environment than conditioned medium from cultured cells. However, considering that at least in plasma, RNase 1 constitutes the majority of ribonucleolytic activity (82), our results position RNase 1 as a likely candidate for producing extracellular tRNA halves and Y RNA fragments *in vivo*. Future studies will have to validate this, as well as address the functional and diagnostic relevance of these extracellular fragments.

## DATA AVAILABILITY

The data reported in this paper were deposited in the Gene Expression Omnibus (GEO) database, www.ncbi.nlm.nih.gov/geo (accession no. GSE148516).

## Supplementary Material

gkaa526_Supplemental_Files
