# Supplementary material for: Processing by RNase 1 forms tRNA halves and distinct Y RNA fragments in the extracellular environment
Source: Nucleic Acids Res. 2020 Jul 1;48(14):8035–49. doi: 10.1093/nar/gkaa526 (PMC7430647; doi:10.1093/nar/gkaa526)
Supplement: gkaa526_Supplemental_Files [file gkaa526_supplemental_files.zip › Supplementary Figures (revision) Nechooshtan.pdf]

A

```
WT          1 ATGGCTCTGGAGAAGTCTCTTGTCCGGCTCCTTCTGCTTGTCCTGATACT   50
              ||||||||||||||||||||||||||||||||||||||||||||||||||
Mutant      1 ATGGCTCTGGAGAAGTCTCTTGTCCGGCTCCTTCTGCTTGTCCTGATACT   50

WT         51 GCTGGTGCTGGGCTGGGTCCAGCCTTCCCTGGGCAAGGAATCCCGGGCCA  100
              ||||||||||||||||||||||||||||||||||||||||||||||||||
Mutant     51 GCTGGTGCTGGGCTGGGTCCAGCCTTCCCTGGGCAAGGAATCCCGGGCCA  100

WT        101 AGAAATTCCAGCGGCAGCATATGGACTCAGACAGTTCCCCCAGCAGCAGC  150
              ||||||||||||||||||||||||||||||||||||||||||||||||||
Mutant    101 AGAAATTCCAGCGGCAGCATATGGACTCAGACAGTTCCCCCAGCAGCAGC  150

WT        151 TCCACCTACTGTAACCAAATGATGAGGCGCCGGAATATGACACAGGGGCG  200
              ||||||||||||||||||||||||||||||||||||||||||||
Mutant    151 TCCACCTACTGTAACCAAATGATGAGGCGCCGGAATATGACACA------  194

WT        201 GTGCAAACCAGTGAACACCTTTGTGCACGAGCCCCTGGTAGATGTCCAGA  250
                           |||||||||||||||||||||||||||||||||||||
Mutant    195 -------------AACACCTTTGTGCACGAGCCCCTGGTAGATGTCCAGA  231

WT        251 ATGTCTGTTTCCAGGAAAAGGTCACCTGCAAGAACGGGCAGGGCAACTGC  300
              ||||||||||||||||||||||||||||||||||||||||||||||||||
Mutant    232 ATGTCTGTTTCCAGGAAAAGGTCACCTGCAAGAACGGGCAGGGCAACTGC  281

WT        301 TACAAGAGCAACTCCAGCATGCACATCACAGACTGCCGCCTGACAAACGG  350
              ||||||||||||||||||||||||||||||||||||||||||||||||||
Mutant    282 TACAAGAGCAACTCCAGCATGCACATCACAGACTGCCGCCTGACAAACGG  331
```

B

```
                         ▼                            ▼
WT          1 KESRAKKFQRQHMDSDSSPSSSSTYCNQMMRRRNMTQGRCKPVNTFVHEP   50
              |||||||||||||||||||||||||||||||||||||   .|:.|...
Mutant      1 KESRAKKFQRQHMDSDSSPSSSSTYCNQMMRRRNMTQ---TPLCTSPW--   45

WT         51 LVDVQNVCFQEKVTCKNGQGNCYKSNSSMHITDCRLTNGSRYPNCAYRTS  100

Mutant     46 --------------------------------------------------   45

                                ▼
WT        101 PKERHIIVACEGSPYVPVHFDASVEDST  128

Mutant     46 ----------------------------   45
```

Supplementary Figure S1. An *RNASE1* mutant generated with CRISPR-Cas9. **(A)** Alignment of the genomic sequence of wild type (WT) *RNASE1* and Sanger sequencing results of the mutant. The start codon is underlined. **(B)** Alignment of the mature protein sequences of wild type and mutant RNase 1. Catalytic residues, as described in (1) are denoted by triangles.

C

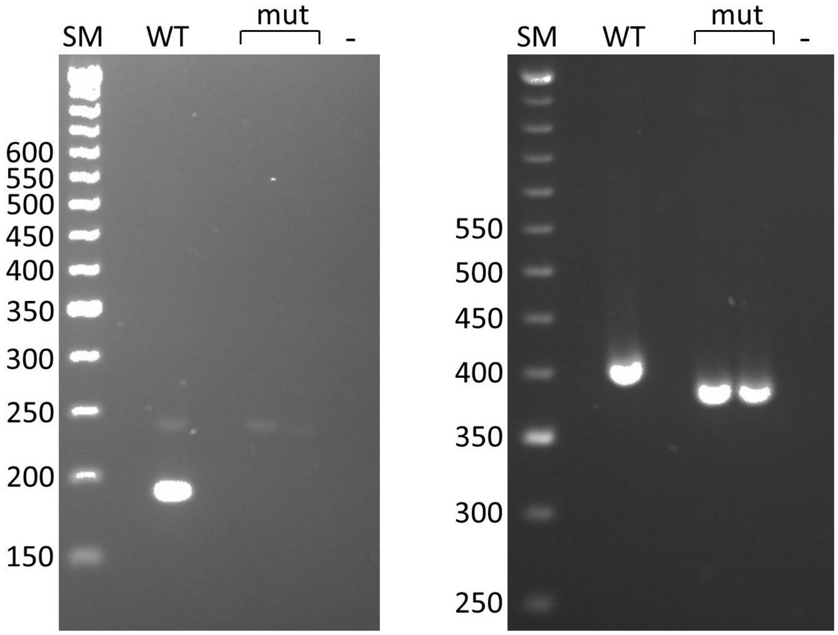

Supplementary Figure S1 (continued). (C) Representative results of PCR for routine verification of cell line identity. Genomic DNA was isolated from respective cultures and used as template. (SM) size marker (bp). (WT) *RNASE1* wild type cells. (mut) *RNASE1* deletion mutant cells. (-) no template control. Left panel- amplification with primer 184 (overlapping deletion) and primer 185 (downstream of deletion). 187 bp product expected in wild type, no product expected in deletion mutant. Right panel- amplification with primer 181 (upstream of deletion) and primer 182 (downstream of deletion). 400 bp product expected in wild type, 381 bp product expected in deletion mutant. PCR primers are detailed in Supplementary Table S1.

D

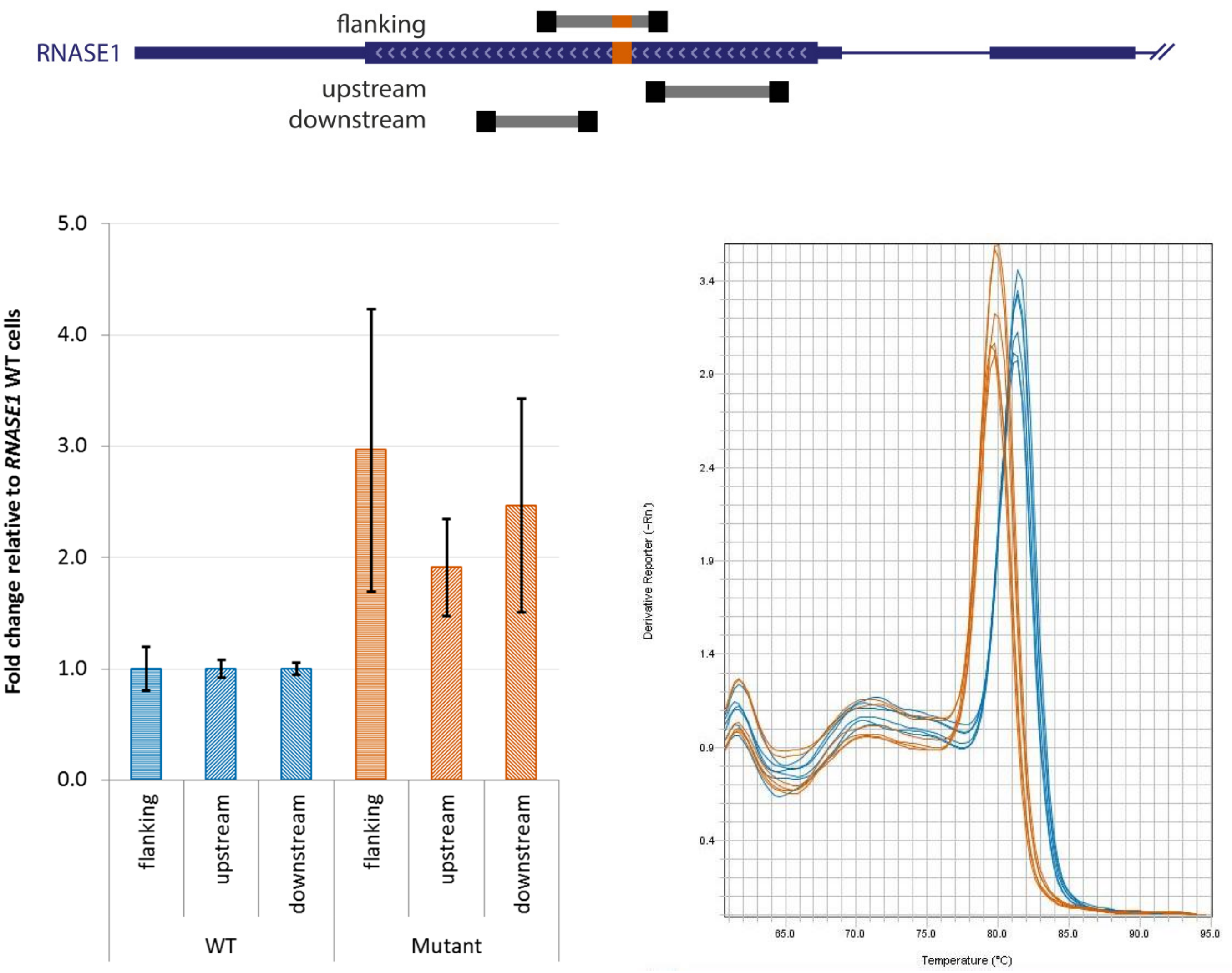

Supplementary Figure S1 (continued). (D) RT-qPCR analysis of expression of *RNASE1 in* wild type and mutant cells. Upper panel- location of primers used. Primers are represented by black boxes, 19 bp deletion is marked in orange. A blue box with arrows denotes the coding sequence of *RNASE1*. Lower left panel- quantitation of *RNASE1* transcripts using the three primer pairs (mean ± SD of three independent replicates). Lower right panel- melt curve of RT-qPCR products synthesized with flanking primers. The difference in melting temperature is indicative of the difference in amplicon size due to the deletion.

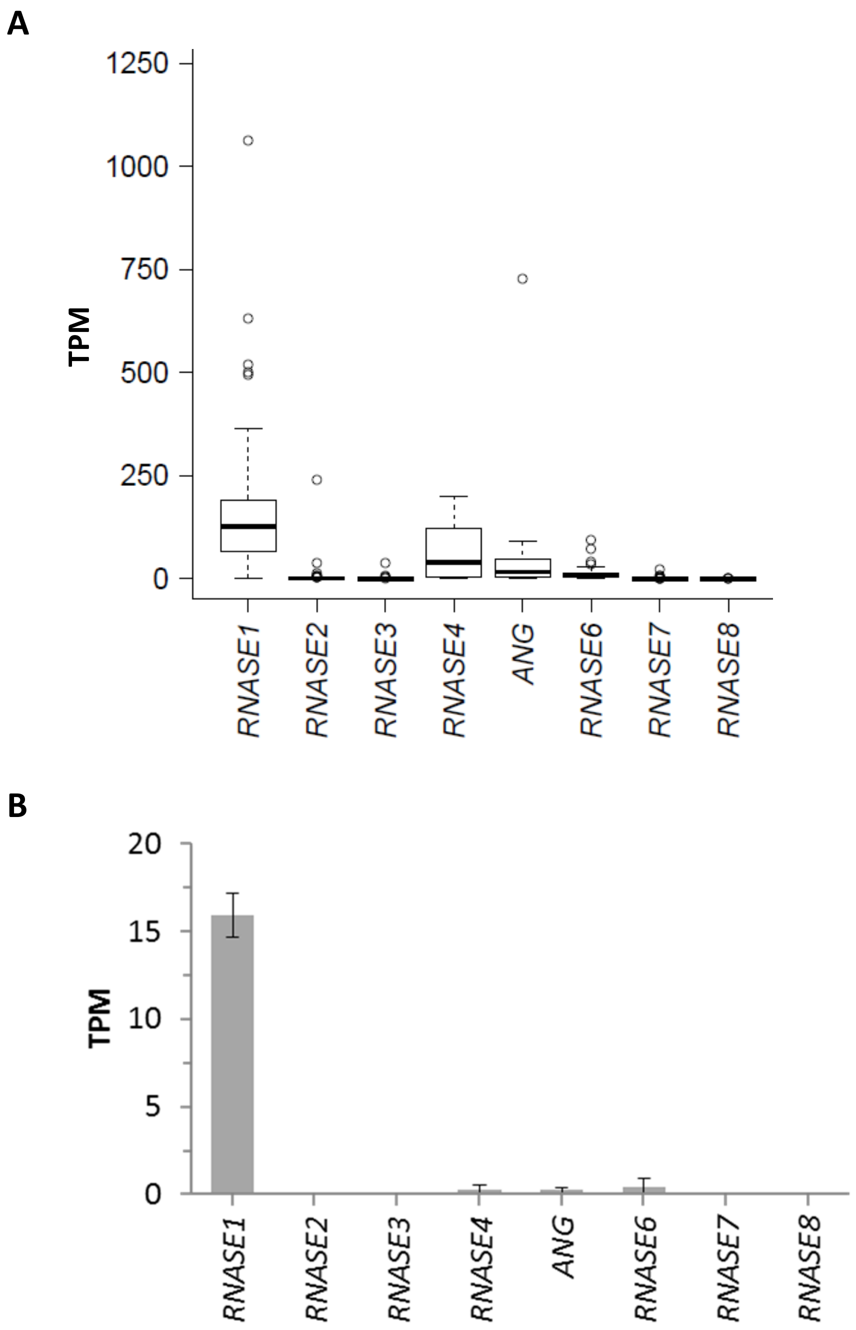

Supplementary Figure S2. Expression profile of pancreatic ribonucleases (RNases). **(A)** Expression of genes encoding catalytically active pancreatic RNases in human tissues. Expression data from 54 different tissues were retrieved from the Genotype-Tissue Expression (GTEx) portal (https://gtexportal.org/home/). For each gene, the median expression value in each of the 54 tissues was obtained. The distributions of these values were plotted as box plots. Boxes represent interquartile range. Horizontal line within each box represents the median of plotted values. Box plots were derived by using R with default parameters. **(B)** Expression levels for genes encoding catalytically active members of the pancreatic RNase family in K562 cells. Nine RNAseq runs were retrieved from ENCODE Project (2) and re-analyzed. (TPM) transcripts per million. Error bars in (B) represent SD.

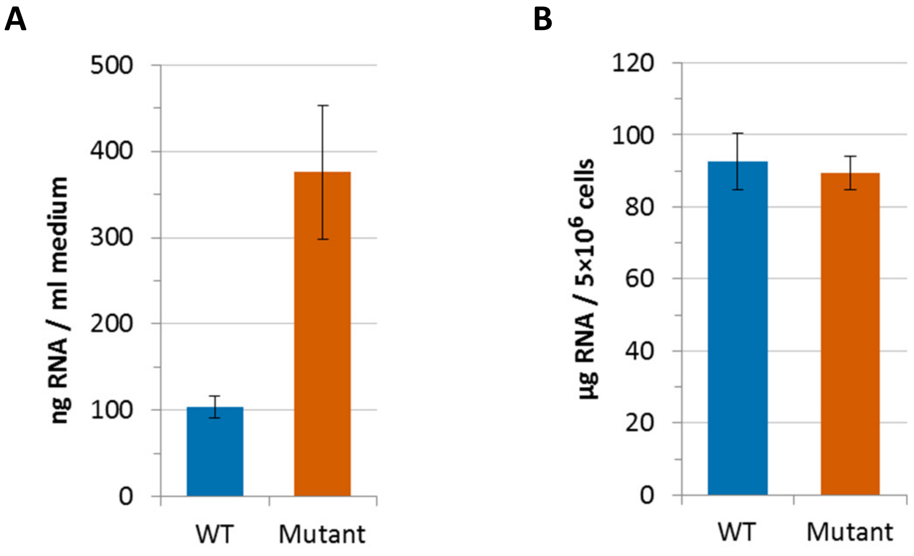

Supplementary Figure S3. RNA yields from non-EV conditioned medium **(A)** and from cells **(B)**. (WT) *RNASE1* wild type source cells. (Mutant) *RNASE1* mutant source cells. Results in each panel represent Mean ± SD of three independent replicates.

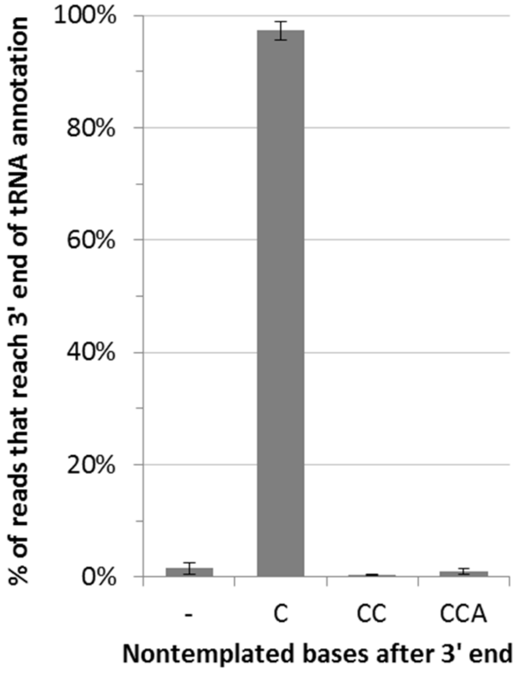

Supplementary Figure S4. Analysis of tRNA 3’ ends in human serum. Datasets of small RNA sequencing of human serum (3) were re-analyzed to determine the status of the nontemplated CCA tail in reads that reach the 3’ end of tRNA gene annotations. Adapter-trimmed reads were required to align to the genome end-to-end, except that non-templated additions of C, CC or CCA were allowed after tRNA gene annotation end. Results represent mean ± SD of six independent replicates.

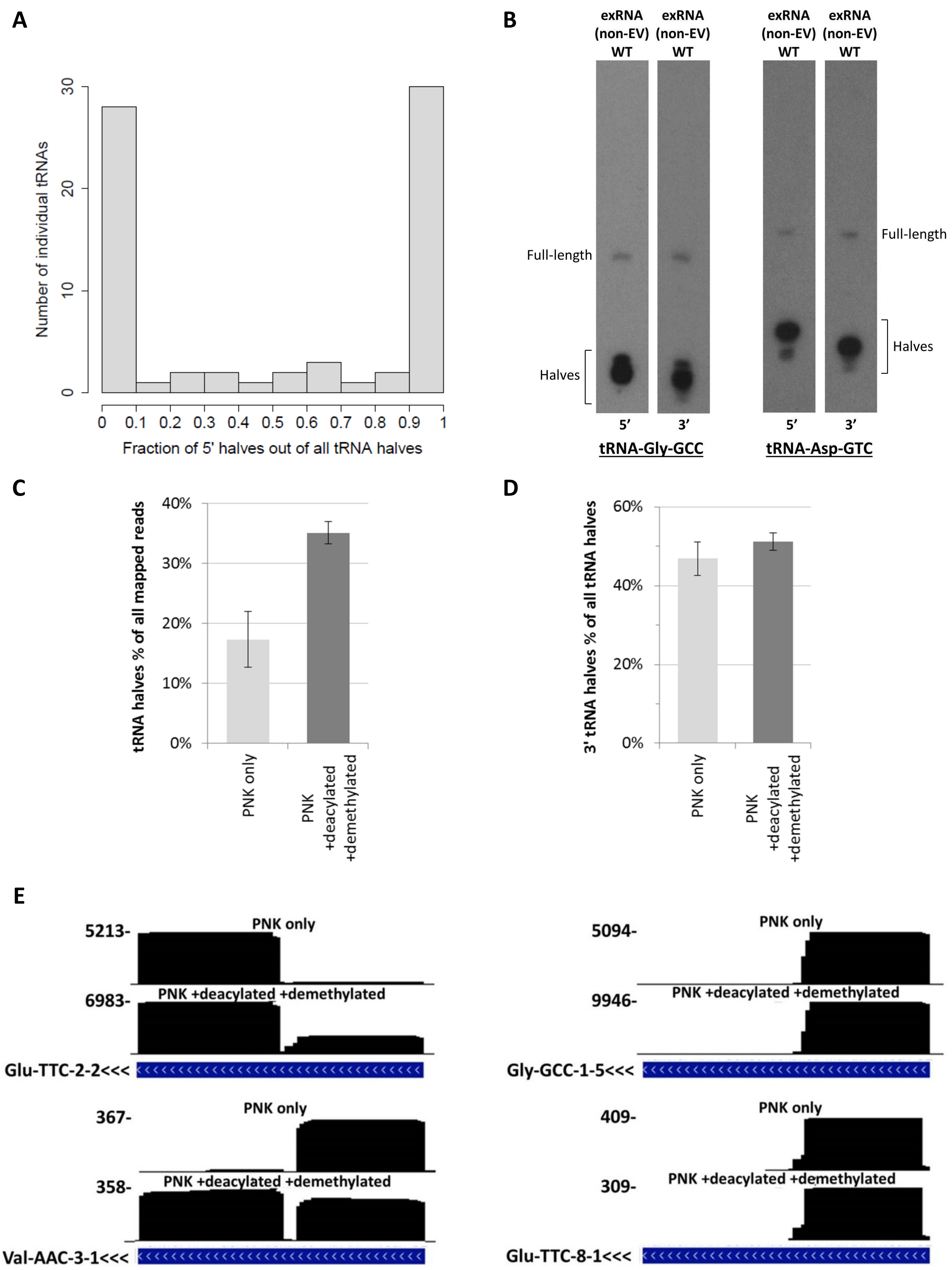
A
Number of individual tRNAs
Fraction of 5' halves out of all tRNA halves
B
exRNA (non-EV) WT
Full-length
Halves
5'
3'
tRNA-Gly-GCC
tRNA-Asp-GTC
C
tRNA halves % of all mapped reads
PNK only
PNK +deacylated +demethylated
D
3' tRNA halves % of all tRNA halves
E
PNK only
PNK +deacylated +demethylated
Glu-TTC-2-2<<<
Gly-GCC-1-5<<<
Val-AAC-3-1<<<
Glu-TTC-8-1<<<

F

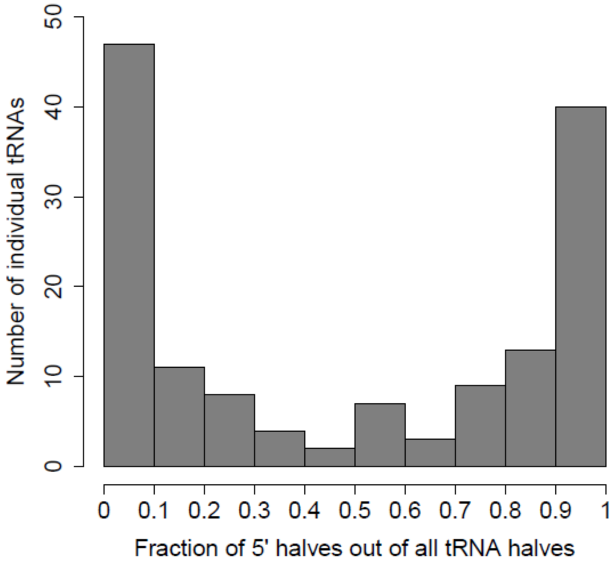

G

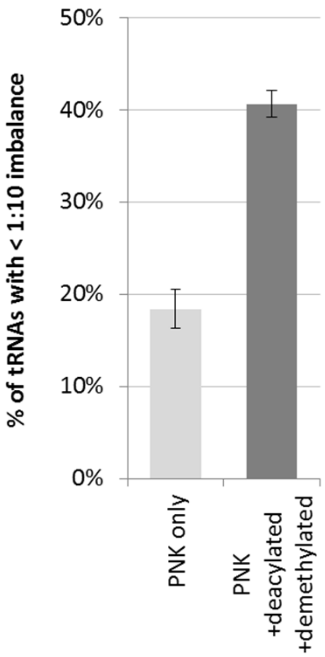

Supplementary Figure S5. 5' half / 3' half imbalance of individual tRNA genes in non-EV exRNA from *RNASE1* wild type cells. **(A)** tRNA genes were grouped by identical mature tRNA sequence. The number of groups (referred to here and in the text as "individual tRNAs") was binned by fraction of 5' halves out of all halves detected for that group on RNAseq. Analysis was limited to groups with more than 50 reads per million tRNA-mapping reads. **(B)** Northern analysis of 5' and 3' halves of tRNA-Gly-GCC and of tRNA-Asp-GTC. These tRNAs show mostly 5' and mostly 3' halves, respectively, on RNAseq. Exposure times were normalized according to the full-length tRNAs, which are detected by probes for both 5' and 3' halves. Note that 3' fragments run shorter than 5' fragments although 3' fragments are longer. This is probably due to stable structures of 3' tRNA halves that are not fully resolved. **(C)** Percent of reads mapping to tRNAs out of all mapped reads. **(D)** Percent of 3' tRNA halves out of all tRNA halves. **(E)** Representative UCSC Genome Browser screenshots of genes affected (left panels) or unaffected (right panels) by addition of 3' deacylation and demethylation of RNA before RNA sequencing. Arrowheads next to gene names denote transcription direction. **(F)** Same analysis as in (A) for RNAseq data from 3' deacylated and demethylated RNA. **(G)** Comparison of results of analyses in panels A and F. tRNAs with less than 1:10 imbalance are those that have 5' half / all halves ratio between 0.1 and 0.9. Data used for producing panels A and F are detailed in Supplementary Table S2. Results in panels C, D and G represent mean ± SD of four independent replicates (PNK only) and two independent replicates (PNK followed by deacylation and demethylation).

A

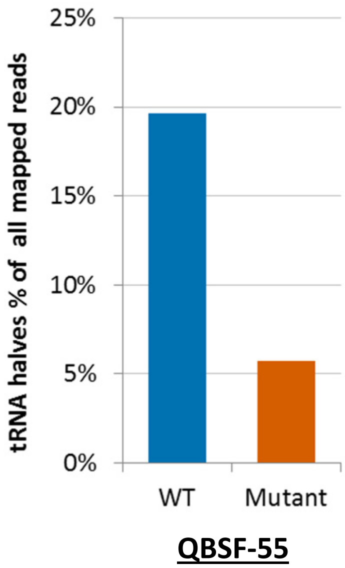

B

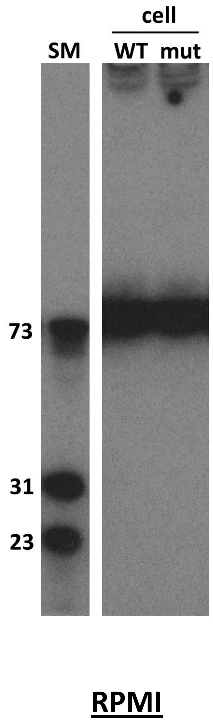

Supplementary Figure S6. Extracellular tRNA halves are formed independently of intracellular stress-induced tRNA halves. (**A**) Percentages of tRNA halves in RNAseq datasets of non-EV exRNA from *RNASE1* wild type (WT) and mutant cells grown in QBSF-55. Compare these results to Figure 2A. QBSF-55 is a serum-free medium that supports long-term growth of K562 cells. **(B)** Long exposure northern blot of cell RNA. Membrane was probed with a probe for 5' halves of tRNA-Glu-CTC. 500 ng total RNA from cells that were used for conditioning of serum-free RPMI was loaded in each lane. (SM) end labeled RNA size marker. (WT) RNA from *RNASE1* wild type cells. (mut) RNA from *RNASE1* mutant cells. Note that sizing is approximate due to incompletely resolved secondary structures.

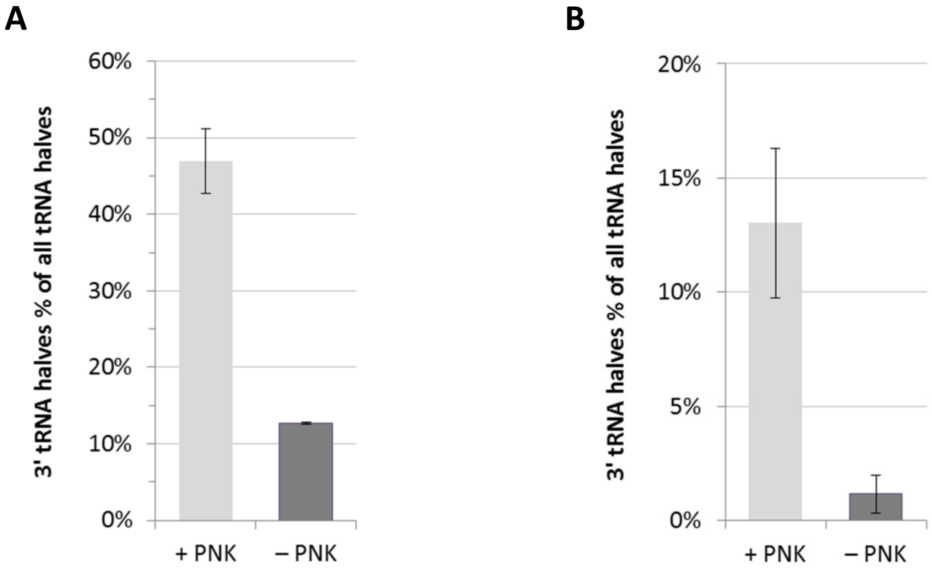

Supplementary Figure S7. Effect of PNK treatment of RNA before sequencing library preparation on 3’ tRNA half representation in RNAseq datasets. **(A)** Percentage of 3’ tRNA halves out of all tRNA halves in small RNA sequencing datasets of non-EV exRNA from *RNASE1* wild type cells. Mean ± SD of four independent replicates and two independent replicates for +PNK and -PNK, respectively. **(B)** Re-analysis of small RNA sequencing datasets of human serum RNA with and without PNK treatment (3). The fraction of 3’ tRNA halves out of all tRNA halves was plotted. Mean ± SD of six independent replicates.
