# Supplementary material for: Processing by RNase 1 forms tRNA halves and distinct Y RNA fragments in the extracellular environment
Source: Nucleic Acids Res. 2020 Jul 1;48(14):8035–49. doi: 10.1093/nar/gkaa526 (PMC7430647; doi:10.1093/nar/gkaa526)
Supplement: gkaa526_Supplemental_Files [file gkaa526_supplemental_files.zip › Supplementary Materials and Methods (revision) Nechooshtan.pdf]

SUPPPLEMENATRY MATERIALS AND METHODS

Quantification of *RNASE1* expression with reverse transcription-quantitative PCR (RT-qPCR)

To measure the expression levels of *RNASE1* in *RNASE1* wild type and mutant cells, $5x10^5$ cells were lysed with 1 ml of TRIzol (Thermo Fisher Scientific) for 5 min. The lysates were mixed with 200 µl of chloroform, incubated at room temperature for 3 min and centrifuged at 12,000 g at 4 °C for 15 min. After centrifugation, 400 µl of the aqueous phase was collected and mixed with 600 µl (1.5 volumes) of 100% ethanol. The resulting mixture was loaded onto RNeasy Mini spin columns (QIAGEN) and purified with an RNeasy Mini Kit (QIAGEN), including on-column treatment of RNA samples with DNase I (QIAGEN) for 1 h, as per recommendations of the manufacturer.

RNA yields were quantified on Nanodrop (Thermo Fisher Scientific) and 1 µg of total RNA was used for reverse transcription (RT) that was primed with 0.5 µg of random hexamer primers. RT was done with ImProm-II Reverse Transcription System (Promega) in the presence of 6 mM $MgCl_2$, as per the manufacturer's protocol. All cDNA samples were diluted 20-fold in nuclease-free water for downstream analyses.

Expression levels of *RNASE1* were measured with quantitative PCR (qPCR) on a 7300 Real Time PCR System (Thermo Fisher Scientific) with the default setup for 20 µl reactions. The assay included the Dissociation Stage to generate melt curves for amplification products. For qPCR, 5 µl of 20-fold diluted cDNA sample were mixed with 5 µl of 3.2 µM primers (Supplementary Table S1), and with 10 µl of Power SYBR Green PCR Master Mix (Thermo Fisher Scientific). Each reaction was performed in triplicate.

Expression levels of *GAPDH* were used for normalization. The delta C_t method was used to determine relative abundance of *RNASE1* transcripts (4). Threshold cycle measurements were done by 7300 System software with the default parameters.

Supplementary Table S1. Oligonucleotides used in this study

Probes for northern analysis

Name	Sequence (DNA)	Target
119	5' CTTAACAATAACCCACAACAC 3'	RNY5 5' side
196	5' AGAATTCTACCACTGAACCACC 3'	tRNA-Gly-GCC 5' half
197<br>198	5' TGCATGGGCCGGGAATC 3'<br>5' TGCATTGGCCGGGAATC 3'	Probe combination for tRNA-Gly-GCC 3' half
199	5' TAACCACTAGACCACCAG 3'	tRNA-Glu-CTC 5' half
200	5' TCACCACTATACTAACGAGGA 3'	tRNA-Asp-GTC 5' half
201	5' GAATCGAACCCCGGTCTCC 3'	tRNA-Asp-GTC 3' half
203	5' CCACACCGGGAGTCGAAC 3'	tRNA-Glu-TTC 3' half
205	5' ACCCAGGACCTTCTGCGT 3'	tRNA-Val-TAC 3' half

Size markers for northern analysis

Name	Sequence (RNA)	Size (nucleotides)
131	5' AGUUGGUCCGAGUGUUGUGGGUU 3'	23
133	5' AGUUGGUCCGAGUGUUGUGGGUUAUUGUUAAGUUGAUUUAAC<br>AUUGUCUCCCCCCACAACCGCGCUUGACUAGCUUGCUGUUU 3'	83
134	5' AGUUGGUCCGAGUGUUGUGGGUUAUUGUUAA 3'	31
155	5' GCUUCUGUAGUGUAGUGGUUAUCACGUUCGCCUCACACGCGA<br>AAGGUCCCCGGUUCGAAACCGGGCAGAAGCA 3'	73

**Primers for PCR**		
Name	Sequence (DNA)	Location in *RNASE1* gene
181	5' CTGGGAAAGTGAGGCCAC 3'	Upstream of deletion
182	5' GGTCCGGTATGCACAGTTG 3'	Downstream of deletion
184	5' GGGCGGTGCAAACCAGTGA 3'	Overlaps 18 out of 19 bases of deletion
185	5' TGGTCCGGTATGCACAGTTGG 3'	Downstream of deletion

**Primers for qPCR**		
Primer name	Sequence (DNA)	Notes
q-GAPDH-F	5′ TGCACCACCAACTGCTTAGC 3′	Primer pair specific for *GAPDH*
q-GAPDH-R	5′ GGCATGGACTGTGGTCATGAG 3′	
q-RNASE1-upstream-F	5′ CGCCTCATCATTTGGTTA 3′	Both primers are upstream of region deleted in *RNASE1* mutant cells
q-RNASE1-upstream-R	5′ CCTTCTGCTTGTCCTG 3′	
q-RNASE1-downstream-F	5′ TGGAGCCGTTTGTCA 3′	Both primers are downstream of region deleted in *RNASE1* mutant cells
q-RNASE1-downstream-R	5′ GCCCCTGGTAGATGT 3′	
q-RNASE1-flanking-F	5′ CCTGCCCGTTCTT 3′	Amplicon includes the region deleted in *RNASE1* mutant cells. Hence, the amplicon in samples from *RNASE1* mutant cells should be shorter than in samples from *RNASE1* wild type cells.
q-RNASE1-flanking-R	5′ ACTGTAACCAAATGATGAG 3′	

sgRNAs for gene disruption		
Name	Sequence	Target
RNASE1_471.16	5' AGGCGCCGGAATATGACACA 3'	*RNASE1* coding sequence
RNASE1_471.17	5' GGCGCCGGAATATGACACAG 3'	*RNASE1* coding sequence
RNASE1_471.58	5' TCGTGCACAAAGGTGTTCAC 3'	*RNASE1* coding sequence
RNASE1_471.59	5' ACCGCCCCTGTGTCATATTC 3'	*RNASE1* coding sequence
RNASE1_471.60	5' TATTCCGGCGCCTCATCATT 3'	*RNASE1* coding sequence
RNASE1_471.67	5' CTGAGTCCATATGCTGCCGC 3'	*RNASE1* coding sequence
Rosa26_(Mm)	5' GAAGATGGGCGGGAGTCTTC 3'	Non-targeting

SUPPLEMENTARY REFERENCES

1. Kover,K.E., Bruix,M., Santoro,J., Batta,G., Laurents,D.V. and Rico,M. (2008) The solution structure and dynamics of human pancreatic ribonuclease determined by NMR spectroscopy provide insight into its remarkable biological activities and inhibition. *J Mol Biol*, **379**, 953-965.
2. Djebali,S., Davis,C.A., Merkel,A., Dobin,A., Lassmann,T., Mortazavi,A., Tanzer,A., Lagarde,J., Lin,W., Schlesinger,F. *et al.* (2012) Landscape of transcription in human cells. *Nature*, **489**, 101-108.
3. Akat,K.M., Lee,Y.A., Hurley,A., Morozov,P., Max,K.E., Brown,M., Bogardus,K., Sopeyin,A., Hildner,K., Diacovo,T.G. *et al.* (2019) Detection of circulating extracellular mRNAs by modified small-RNA-sequencing analysis. *JCI Insight*, **4**, e127317.
4. Pfaffl,M.W. (2001) A new mathematical model for relative quantification in real-time RT-PCR. *Nucleic Acids Res*, **29**, e45.
